# PhcX Is a LqsR-family response regulator that contributes to *Ralstonia solanacearum* virulence and regulates multiple virulence factors

**DOI:** 10.1128/mbio.02028-23

**Published:** 2023-10-03

**Authors:** Qingmei Liu, Chuhao Li, Xiaohan Zhang, Mengfan Ding, Xinyue Liao, Jinli Yan, Ming Hu, Leilei Yang, Xiaoqing Wang, Lisheng Liao, Peng Li, Xiaofan Zhou

**Affiliations:** 1 Guangdong Laboratory for Lingnan Modern Agriculture, Guangdong Province Key Laboratory of Microbial Signals and Disease Control, Integrative Microbiology Research Centre, South China Agricultural University, Guangzhou, China; 2 School of Agricultural Science, Xichang University, Xichang, China; 3 Ministry of Education Key Laboratory for Ecology of Tropical Islands, Hainan Provincial Key Laboratory for Tropical Plant and Animal Ecology, College of Life Sciences, Hainan Normal University, Haikou, China; Institut Pasteur, Paris, France

**Keywords:** *Ralstonia solanacearum*, response regulator, virulence factors, quorum sensing, Phc, Lqs

## Abstract

As one of the most notorious and successful phytopathogenic bacteria, *Ralstonia solanacearum* controls the transition between long-term survival and pathogenic modes through an intricate regulatory network, the understanding of which remains incomplete despite years of effort. In this study, we identified PhcX, a previously uncharacterized response regulator in *R. solanacearum*, and uncovered its essential functions in modulating virulence and metabolism. The *phcX* deletion mutant exhibited substantial phenotypic alterations, including slower initial growth, altered response to host extract, reduced motilities, polygalacturonase activity, and exopolysaccharide production, elevated biofilm formation, delayed hypersensitive response, and impaired virulence. Moreover, ~16% of all genes were differentially expressed in the mutant, among which the genes associated with virulence, nitrogen metabolism, and regulation were overrepresented (e.g., most T3SS/T3Es genes). Many of these traits and genes were regulated by PhcX and the global virulence regulator PhcA, but 81.4% of the genes were regulated in opposite directions. The functions of PhcX were largely conserved in *R. solanacearum* EP1 and GMI1000 strains. Apparent orthologs of PhcX are widely distributed in Proteobacteria, including the LqsR quorum sensing (QS) response regulator in *Legionella pneumophilia*. Notably, our data suggest that *phcX* was originally part of the Lqs QS system but was decoupled from Lqs in *Ralstonia*/*Cupriavidus*, physically linked to the *phc* QS genes, and connected with the virulence regulatory network in *Ralstonia* during its evolution. The findings of this study contribute to a better understanding of the virulence and metabolism regulation mechanisms in *R. solanacearum* and shed light on the evolution of its complex regulatory network.

**IMPORTANCE:**

The bacterial wilt caused by the soil-borne phytopathogen *Ralstonia solanacearum* is one of the most destructive crop diseases. To achieve a successful infection, *R. solanacearum* has evolved an intricate regulatory network to orchestrate the expression of an arsenal of virulence factors and fine-tune the allocation of energy. However, despite the wealth of knowledge gained in the past decades, many players and connections are still missing from the network. The importance of our study lies in the identification of PhcX, a novel conserved global regulator with critical roles in modulating the virulence and metabolism of *R. solanacearum*. PhcX affects many well-characterized regulators and exhibits contrasting modes of regulation from the central regulator PhcA on a variety of virulence-associated traits and genes. Our findings add a valuable piece to the puzzle of how the pathogen regulates its proliferation and infection, which is critical for understanding its pathogenesis and developing disease control strategies.

## INTRODUCTION

*Ralstonia solanacearum* is a soil-borne plant pathogen with a broad host range and global distribution. The bacterial wilt disease caused by *R. solanacearum* species complex (RSSC) usually results in serious losses in the yields of hundreds of plant species all over the world, including numerous important economic crops such as tomato, potato, tobacco, and pepper (1, 2). According to the EPPO Global Database, new host plants and geographical distributions are constantly being reported for RSSC, posing an ever-increasing threat to agroecosystems worldwide. *R. solanacearum* adjusts its metabolic and pathogenic properties through sophisticated and complicated regulatory systems to accommodate different environments and pathogenic processes. A better understanding of the key regulators and regulatory mechanisms during its pathogenic progress is critical for developing successful control strategies.

The RSSC infection process begins with attachment to host roots. At the initial infection stage, the pathogen senses and moves toward root exudates using chemotaxis (3) and flagellar motility (4, 5) attaches to the plant roots via polysaccharides (6), adhesin proteins (7 – 9), and cell-surface appendages such as pili (10 – 12). Besides, the *hrpB* and *hrpG* regulatory genes are also required for the root infection process (13). After entering the host through wounds, *R. solanacearum* multiplies to high population densities, infects the cortex, and colonizes the xylem, which mainly requires the secretion of cell wall-degrading enzymes (14) and exopolysaccharides (EPS) (15). As a result of pathogen proliferation and EPS accumulation, infected plants develop wilting symptoms and eventually die (16). Multiple other factors also contribute to the pathogenicity of *R. solanacearum*, including biofilms (8, 17), ralfuranones (18), type III effectors (T3Es) (19, 20), nitrate assimilation (21), and cbb3-type cytochrome c oxidase (22). As a soil-borne and vascular pathogen, *R. solanacearum* modulates virulence determinants during transmission between saprophyte and pathogen through intricate virulence regulatory networks.

The Phc quorum sensing (QS) system is at the center of the regulatory network modulating virulence in *R. solanacearum*. The Phc QS system consists of the QS signal sensory cascade phcBSRQ (23, 24) and the global transcriptional regulator PhcA (25). PhcB is a methyltransferase that synthesizes the QS signal methyl 3-hydroxypalmitate or methyl 3-hydroxymyristate (3-OH MAME) (26). PhcS/R is a two-component system, of which PhcS is a sensor histidine kinase, and PhcR acts as a response regulator containing both the sensor histidine kinase and a receiver domain. PhcQ is a recently identified regulator protein with a receiver domain similar to the receiver domain of PhcR (27). PhcBSRQ is encoded by an operon and works together to sense the concentration of QS molecules and regulate many QS-dependent genes (28).

PhcA is a global regulator that orchestrates the expression of a wide range of genes in response to cell density; it positively regulates the expression of genes mainly expressed at high cell density, such as those related to EPS and lipopolysaccharide (LPS) biosynthesis, cellulase, ralsolamycin and ralfuranone biosynthesis, adhesion, biofilm formation, and type VI secretion system (T6SS), while it negatively regulates the genes highly expressed at low cell density, such as those underlying swimming and twitching motility, chemotaxis, type III secretion system (T3SS), polygalacturonase, siderophore staphyloferrin B, and nitrate reductase (4, 29, 30). PhcA also plays a critical role in regulating the metabolism of *R. solanacearum* to achieve optimal resource allocation (31, 32). Fine-tuned regulation and coordinated expression of genes underlying virulence and metabolism are key to the success of *R. solanacearum* in survival and infection (33, 34). Recent studies have identified additional master regulators of both virulence and metabolism, such as EfpR (35, 36), and further highlighted the importance of interaction between the two processes.

Over the years, multiple other virulence regulators have been characterized in *R. solanacearum*, such as *solR* and *rasR* (acyl-HSL responsive regulon) (37), *hrpB/hrpG* (T3SS and other pathogenicity functions) (38, 39), *xpsR* and *epsR* (EPS biosynthesis) (40, 41), *pehSR* (polygalacturonase production and other virulence functions) (42), and *flhDC* (flagellar motility) (43). Furthermore, ralfuranones, a lectin protein, LecM, and EPS I have all been demonstrated to participate in the feedback of the QS signaling pathway (44, 45). Although much progress has been made, there are still many missing pieces in the regulatory network of *R. solanacearum*, and new players continue to be discovered. For instance, anthranilic acid and its response regulator, RaaR, were recently reported to control important biological functions of *R. solanacearum* and the synthesis of QS signals, including 3-OH MAME and AHL signals (46, 47). A putative sensor histidine kinase, PhcK, was found to be a positive regulator of *phcA* in *R. solanacearum* OE1-1 (48).

In this study, we set out to screen for additional virulence regulators in *R. solanacearum* by conducting *in vitro* continuous passage experiments and obtaining an EPS-deficient mutant. Whole-genome sequencing of the mutant identified a mutation of the gene *RSc2734* encoding a putative response regulator orthologous to *Legionella* LqsR. *RSc2734* has an RSSC-wide conserved genomic location adjacent to the *phcBSRQ* operon, thus being designated as *phcX*. We generated *phcX* deletion mutants in both *R. solanacearum* EP1 and GMI1000, conducted growth assays, phenotypic characterization, pathogenicity tests, and transcriptome analyses, and compared the regulons of PhcX and PhcA. Our results suggest that the PhcX is a novel global regulator in *R. solanacearum* with broad regulatory functions in both virulence and metabolism.

## RESULTS

### Identification of a putative response regulator, PhcX, from spontaneous EPS-deficient mutants of *R. solanacearum* EP1

We conducted *in vitro* continuous passage experiments in *R. solanacearum* strain EP1 and screened for EPS-deficient mutants at each passage using visible EPS production on the solid medium as the indicator. One mutant from the 11th generation and two mutants from the 23rd generation were obtained and sequenced (Table S1A). The two EPS-deficient mutants from the 23rd generation contained the same missense mutations in *pilT* (*AC251_RS04265*) and the gene *AC251_RS03885* as determined by whole-genome sequencing analysis (Fig. S1a). According to the NCBI RefSeq database, the *AC251_RS03885* gene encodes a putative response regulator (RefSeq protein id: WP_011002641.1). AC251_RS03885 was predicted to contain no signal peptide or transmembrane domain and have a cytoplasmic localization. Despite its low levels of sequence similarity to other proteins in EP1, AC251_RS03885 carries a response regulator receiver domain (InterPro id: IPR001789) also found in PhcR and PhcQ. Information from the STRING database (49) indicated that AC251_RS03885 is strongly associated with the Phc QS system and other virulence regulators (e.g., VsrB and RcsC) (Fig. S1b). In the EP1 genome, the AC251_RS03885 gene is located immediately upstream of the *phcBSRQ* operon, with a 596-bp intergenic region in between. Notably, our analysis of the 517 RSSC genomes in the NCBI RefSeq database showed that only 2 lacked AC251_RS03885 (Table S2). Moreover, in strains containing *phcX*, this gene was located near the known *phc* cluster, although its location relative to the other *phc* genes varied by strain as this locus appears to have undergone rearrangements in many RSSC genomes. The AC251_RS03885 gene was thus named *phcX* due to its close proximity to the *phc* genes.

### Deletion of *phcX* impairs the growth rate of EP1 during the early proliferation stage and alters the response to ground and filtered tomato stems

**FIG 1:**
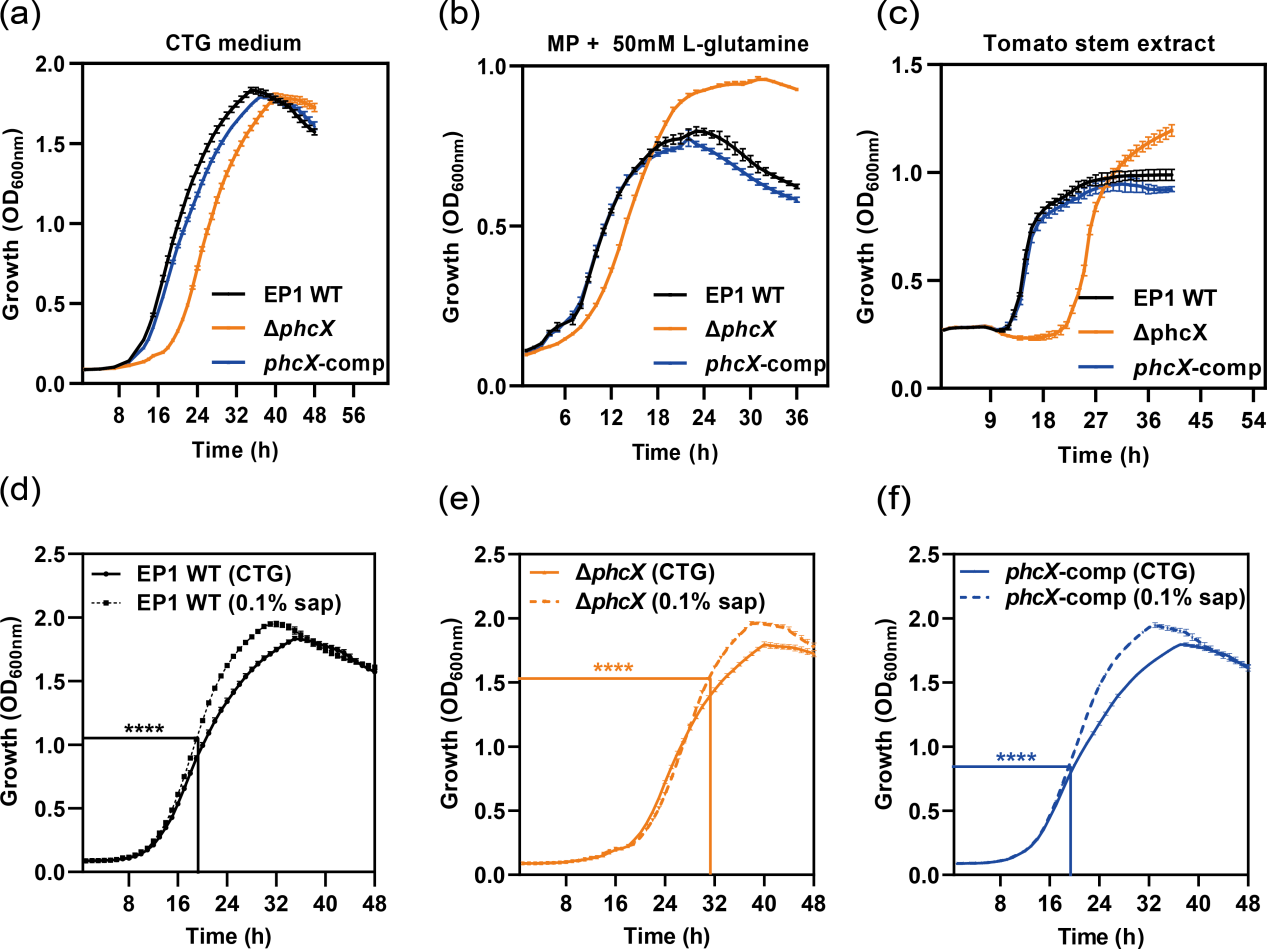
Growth curves of *R. solanacearum* wild-type EP1, Δ*phcX*, and *phcX*-comp strains. (**a**) Growth curves in CTG medium. (**b**) Growth curves in MP medium with L-glutamine to a final concentration of 50 mM. (**c**) Growth curves in ground and filtered tomato stems. Growth curves of wild-type EP1 (**d**), Δ*phcX* (**e**), and *phcX*-comp (**f**) in CTG medium and CTG medium with ground and filtered tomato stems to a final concentration of 0.1% (vol/vol). Rectangular lines in panels **d**, **e**, and **f** show the point where each strain started to exhibit significantly higher cell density in CTG medium with 0.1% tomato xylem sap than in pure CTG medium. The experiments were repeated three times, with five technical replicates per experiment. The data were presented as mean ± standard deviation. Asterisks indicate that the cell density values at the same time point were significantly different (****P < 0.0001, multiple unpaired *t*-test).

In order to investigate the function of PhcX and reveal its relationship with the Phc QS system, the *phcX* gene deletion mutant (Δ*phcX*) and its complementation strain (*phcX*-comp) were constructed from strain EP1 (Table S1B). The growth curve assay was conducted on wild-type EP1, Δ*phcX*, and *phcX*-comp under three different nutrition conditions. Compared with wild-type EP1, Δ*phcX* showed a lower growth rate at the early proliferation stage as it took a longer time for Δ*phcX* to reach an OD_600_ of 0.3 in the CTG medium (a rich medium; delayed by ~6 h; Fig. 1a), the MP medium (39) (a minimal, nutrient-poor medium) containing 50 mM L-glutamine (delayed by ~2–3 h; Fig. 1b), and pure ground and filtered tomato stems (mimicking the *in vivo* nutrition condition; delayed by >9 h; Fig. 1c). We calculated the growth rates of strains in CTG medium and found that Δ*phcX* had a much longer lag phase duration (13.36 h) and a slightly lower exponential growth rate (0.20 h^−1^) than wild-type EP1 (7.82 h; 0.22 h^−1^) and *phcX*-comp (8.64 h; 0.22 h^−1^) (Fig. S2). In the ground and filtered tomato stems, the lag-phase of Δ*phcX* (21.67 h) was also substantially prolonged compared with wild-type EP1 (11.92 h) and *phcX*-comp (11.84 h) (Fig. S2). Interestingly, Δ*phcX* entered the stationary phase at a similar cell density as the wild-type EP1 in the rich medium (Fig. 1a) but could grow to higher OD_600_ values in the minimal medium (Fig. 1b) and ground and filtered tomato stems (Fig. 1c). The growth curves of *phcX*-comp and the wild-type strain EP1 were similar under all three conditions.

Our results revealed a more pronounced difference in the growth curves of the wild-type EP1 and Δ*phcX* in ground and filtered tomato stems (Fig. 1c). To further investigate the role of *phcX* in the response of EP1 to ground and filtered tomato stems, we also examined the growth curves of the EP1 strains in CTG medium containing 0.1% ground and filtered tomato stems. Our results showed that, for each strain, adding 0.1% sap did not alter the lag phase growth curve but led to an increased log phase growth rate and a higher maximum cell density (Fig. 1d through f). Meanwhile, with the addition of 0.1% sap, the wild-type EP1 and *phcX*-comp started to exhibit significantly higher cell density at an earlier time point in the log phase (at the 19th h; *t* test, *P* < 0.0001; Fig. 1d and f) than Δ*phcX* (at the 31st h, *t* test, *P* < 0.0001; Fig. 1e). Therefore, the deletion of *phcX* delayed the induction of EP1 growth rate by stem sap. Overall, our results suggest that *phcX* plays a role in the early proliferation of EP1 and its response to host plant extract. Reverse transcription-quantitative polymerase chain reaction (RT-qPCR) analyses showed that genes related to nitrogen metabolism (*narG*/*H*/*L*) and cytochrome c oxidase (*CcoO*/*N*/*P*) were down-regulated in Δ*phcX* compared with the wild-type (Table S3A).

### Multiple virulence-related phenotypes of EP1 are modulated by PhcX

We examined Δ*phcX* for several virulence-related phenotypes, including swimming and twitching motilities, polygalacturonase and cellulase activities, biofilm formation, EPS production, and hypersensitive response (HR).

#### Motility

**FIG 2:**
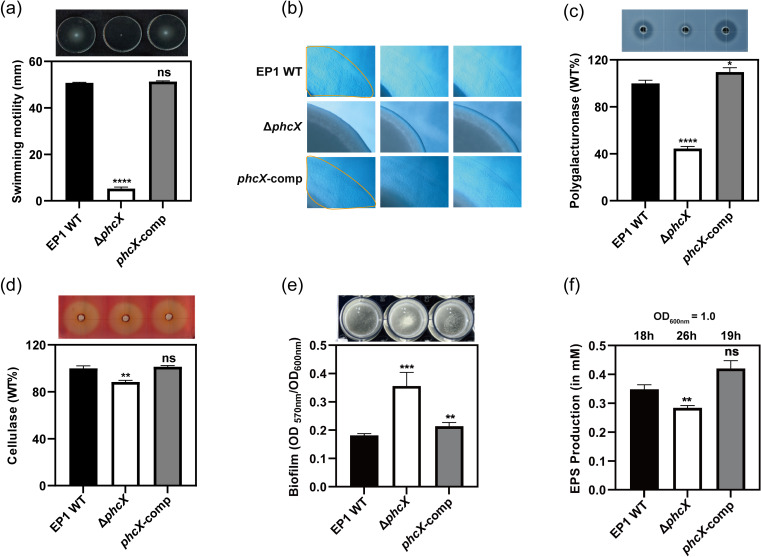
Phenotypic characterization of *R. solanacearum* wild-type EP1, Δ*phcX*, and *phcX*-comp. The phenotypes examined include (**a**) swimming motility, (**b**) twitching motility, (**c**) polygalacturonase activity, (**d**) cellulase activity, (**e**) biofilm formation, and (**f**) quantitative measurements of EPS production. Swimming and twitching motility assays were conducted on 0.3% and 1.6% agar plates, respectively. Representative optical microscope images (4× magnification) of twitching motility assays were taken. Polygalacturonase and cellulase activities were detected on bioassay plates, and the final results of Δ*phcX* and *phcX*-comp were normalized to those of the wild-type EP1, which was set to a value of 100% for comparison. Biofilm formation was assessed in 24-well polystyrene plates, quantified with absorbance at 570 nm, and normalized with the cell density as measured by OD_600_. EPS production in the culture broth supernatant of *R. solanacearum* cells at an OD_600_ of 1.0 was determined by the Elson-Morgan method, and results were shown in the concentration of N-acetyl-galactosamine in mM. The numbers (in h) on top of panel (**f**) indicate the time it took for each strain to reach an OD_600_ of 1.0 from an initial OD_600_ of 0.01. All experiments were repeated at least three times, with at least three technical replicates per experiment. The data were presented as mean ± standard deviation. Asterisks indicate values significantly different from those of wild-type EP1 (ns, no significance; **, *P* < 0.01; ***, *P* < 0.001; ****, *P* < 0.0001, unpaired *t*-test).

Swimming and twitching motilities are important virulence factors of RSSC required for plant colonization and full virulence, especially during the early infection stages (10, 43). Here, the swimming and twitching motilities of the EP1 strains were compared. The deletion of *phcX* substantially decreased the swimming and twitching motilities relative to the wild-type EP1, and the complementation of *phcX* fully restored the corresponding phenotypes (Fig. 2a and b). RT-qPCR tests demonstrated that the expression levels of genes related to swimming motility (e.g., *fliC*, *flgK*, *flgN*) were reduced in Δ*phcX* compared with the wild-type (Table S3A).

#### Plant cell wall degrading enzymes

Plant cell wall degrading enzymes are key virulence factors contributing to the virulence of *R. solanacearum* (50, 51). Here, the polygalacturonase and cellulase activities of the EP1 strains were analyzed. Results indicated that Δ*phcX* exhibited substantially attenuated polygalacturonase activity (*P* < 0.0001) and slightly but significantly decreased cellulase activity (*P* < 0.01) compared with the wild-type EP1 and *phcX*-comp (Fig. 2c and d). An RT-qPCR test of the polygalacturonase-related gene *pehB* showed that it was down-regulated in Δ*phcX* (Table S3A).

#### Biofilm

The polyvinyl chloride crystal violet assay and pellicle formation assay were used to quantify *in vitro* bacterial adhesion to surfaces and cohesion, respectively. The results showed that the adherent biofilm production of Δ*phcX* was substantially higher (*P* < 0.001) than that of the wild-type EP1 (Fig. 2e). In addition, after 36 h of static culture in CTG medium, Δ*phcX* formed considerably more floating pellicles at the air-liquid interface than the wild-type EP1 (Fig. 2e). *phcX*-comp produced slightly more adherent biofilm (*P* < 0.01) than the wild-type EP1 and showed no significant difference in pellicle formation (Fig. 2e). Furthermore, *lecM*, encoding a lectin, is reported to be involved in the biofilm formation in *R. solanacearum* (8) and was found to be significantly up-regulated in Δ*phcX* by our RT-qPCR assay (Table S3A).

#### EPS

In bacterial wilt diseases, the accumulation of pathogen-produced EPS is a major factor that blocks the water traffic within xylem vessels and ultimately causes plant wilting symptoms (25). The EPS production of the EP1 strains was analyzed by an adapted Elson and Morgan assay for EPS quantification in *R. solanacearum* (52). Each strain was inoculated at an OD_600_ of 0.01 and cultured until an OD_600_ of 1.0, and the EPS accumulated during the culturing process was measured. Δ*phcX* was cultured for 26 h in the experiment, which was ~40% longer than the wild-type EP1 (18 h) and *phcX*-comp (19 h). However, Δ*phcX* produced 18% less EPS than the wild-type EP1 (*P* < 0.01), while *phcX*-comp did not show significantly different EPS production from the wild-type (Fig. 2f). The EPS repressor gene *epsR* was up-regulated in Δ*phcX* according to RT-qPCR analysis (Table S3A).

#### HR

**FIG 3:**
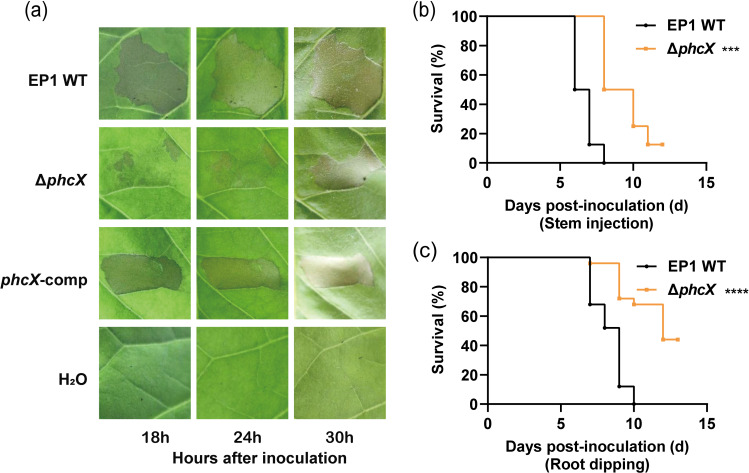
HR development and virulence of *R. solanacearum* strains. HR development (**a**) induced by *R. solanacearum* wild-type EP1, Δ*phcX*, and *phcX*-comp was tested on tobacco leaves. Photographs were taken at the indicated times after inoculation. Double-distilled H_2_O was used as the negative control. Tomato plants were inoculated with *R. solanacearum* wild-type EP1 and Δ*phcX* via stem injection (**b**) and root dipping (**c**) methods. For stem injection, a 2-µL bacterial suspension was inoculated into the stem wound created by toothpicks. For root dipping inoculation, tomato seedlings were soaked in bacterial suspensions (OD_600_ of 1.0) for 30 min and then exposed to air for 15 min on dry paper. The number of dead plants was recorded daily, and Kaplan-Meier survival analysis of plants inoculated with *R. solanacearum* strains was carried out (***, *P* < 0.001; ****, *P* < 0.0001, Gehan-Breslow-Wilcoxon test).

T3SS and T3Es are major pathogenicity determinants required for the full virulence of RSSC and can induce a strong defense response often associated with an HR (20). To determine if the deletion of *phcX* affects the ability of EP1 to elicit HR, we inoculated the EP1 strains on tobacco leaves and monitored the development of necrotic lesions. The results showed that tobacco leaves inoculated with Δ*phcX* developed necrotic lesions much slower than those inoculated with the wild-type EP1 or *phcX*-comp, particularly at the early time points (18 and 24 h) (Fig. 3a), and our RT-qPCR results also revealed that several genes encoding T3SS components (*hrcC*, *hrpB*, *hrpG*) and HR elicitor (*popA*) in Δ*phcX* were down-regulated (Table S3A).

### *phcX* deletion impairs the virulence of EP1

As the deletion of *phcX* affected various virulence-related phenotypes of EP1, we inoculated tomato cultivar Zhongshusihao with the EP1 strains by stem injection and root dipping methods to assess the role of *phcX* in virulence. In the stem injection assay, tomato plants inoculated with Δ*phcX* wilted significantly slower than those inoculated with wild-type EP1 (*P* < 0.001, Fig. 3b). A similar result was also obtained by using the dipping method (*P* < 0.0001, Fig. 3c). In addition, all the plants inoculated with wild-type EP1 died within 2 weeks, whereas 12.5% and 44% of the plants inoculated with Δ*phcX* survived in the stem injection and root dipping assays, respectively. Together, our results suggest that *phcX* is important for the full virulence of EP1.

### *phcX* deletion leads to broad down-regulation of genes underlying motility, chemotaxis, virulence, and nitrogen metabolism

To further investigate the regulatory roles of *phcX*, RNA-seq analysis of wild-type EP1 and Δ*phcX* (both cultured in CTG medium until an OD_600_ of 0.3) was conducted. We obtained 7.5 to 7.9 million high-quality read pairs per sample from the wild-type and Δ*phcX* samples, mapped quality-filtered reads of each sample to the EP1 genome, and performed differential gene expression analysis. Principal component analysis (PCA) indicated high reproducibility among biological replicates (Fig. S3a). Compared with the wild-type, 845 differentially expressed genes {DEGs; false discovery rate (FDR) <0.05, |log_2_[fold change (FC)]| ≥1} were identified in Δ*phcX*, among which 473 and 372 genes were up- and down-regulated, respectively (Table S3B). The results of RNA-seq analysis were validated by RT-qPCR assays on selected genes, which revealed similar differential expression patterns (Table S3A).

To reveal the functional types of genes regulated by *phcX*, we performed Gene Ontology (GO) and Kyoto Encyclopedia of Genes and Genomes (KEGG) pathway enrichment analyses on DEGs. GO terms significantly enriched (FDR <0.05) among down-regulated genes include molecular transducer activity, cytoskeletal motor activity, organelle, extracellular region, external encapsulating structure, cell motility, programmed cell death, signaling, and nitrogen cycle metabolic processes (Table S3C). The KEGG analysis (FDR <0.05) showed that the flagellar assembly, bacterial chemotaxis, two-component system, and nitrogen metabolism pathways were most enriched among down-regulated genes, while the steroid degradation pathway was enriched among up-regulated genes (Table S3D). These enriched GO terms and KEGG pathways are largely consistent with the phenotypes of Δ*phcX*.

**FIG 4:**
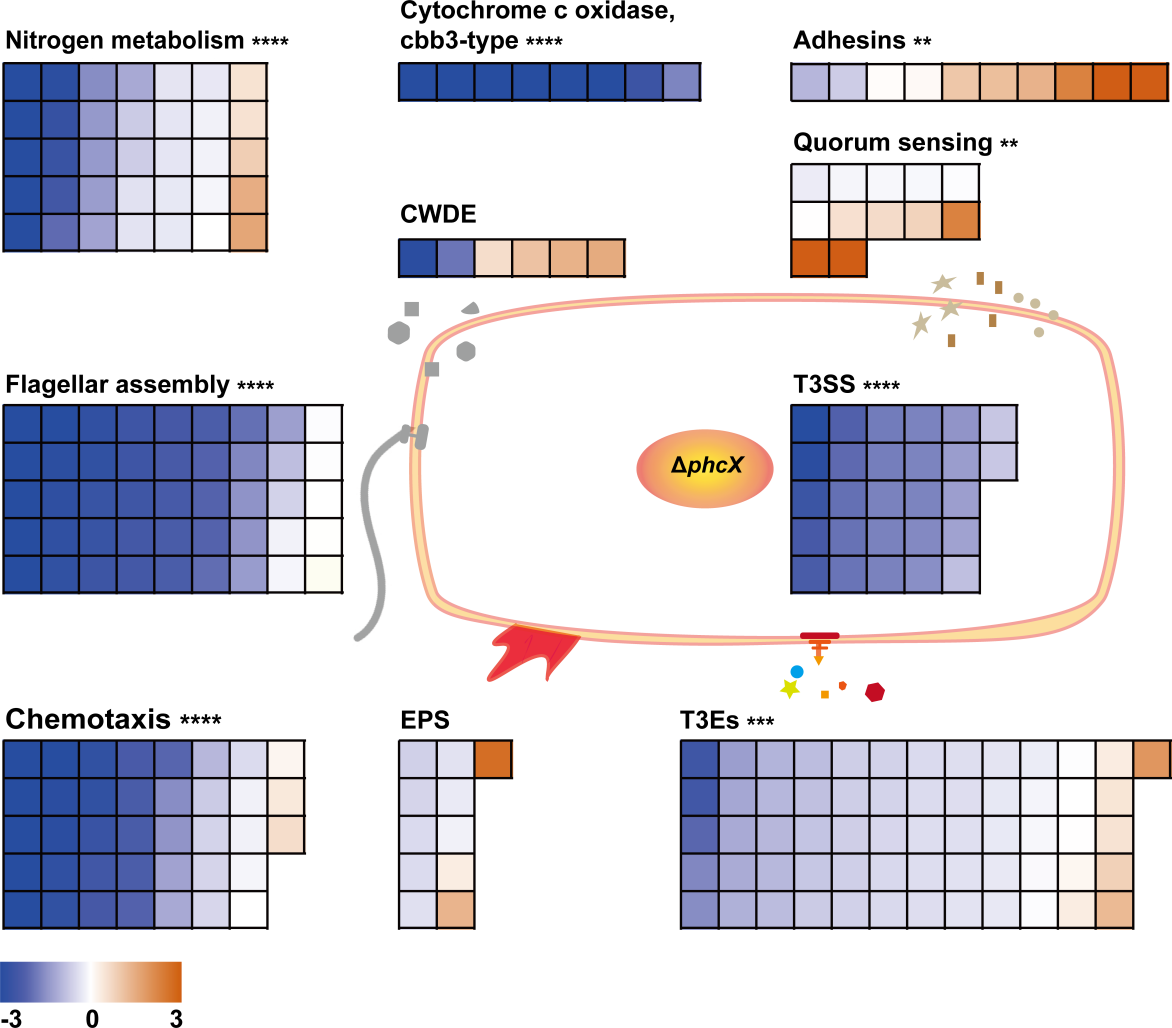
Differential expression patterns of genes involved in various virulence-related functional categories in Δ*phcX*. GSEA was carried out based on the gene sets of *R. solanacearum* compiled from multiple sources and the differential gene expression data generated in the RNA-seq analysis of the wild-type EP1 and Δ*phcX* (both cultured in CTG medium until an OD_600_ of 0.3). Small squares corresponded to genes in each category and were colored based on their log_2_ FC values (blue, log_2_ FC <0; yellow, log_2_ FC >0). Asterisks indicate significant GSEA results (**, *q* < 0.01; ***, *q* < 0.001; ****, *q* < 0.0001).

To further understand PhcX-mediated gene regulation, we compiled gene sets (Table S3E) associated with various biological processes and molecular machinery, particularly those related to virulence, and conducted gene set enrichment analysis (GSEA). As shown in Fig. 4, there is a significant enrichment and global down-regulation of genes associated with nitrogen metabolism (FDR = 5.26 × 10^−7^) and cbb3-type cytochrome c oxidases (FDR = 6.87 × 10^−7^), chemotaxis (e.g., *cheY*, *cheA*, *cheW*; FDR = 6.0 × 10^−10^), flagellar assembly (e.g., *motA*, *motB*, *flgK*, *fliM*; FDR = 6.0 × 10^−10^), T3SS (e.g., *hrpB*, *hrpG*, *hrcC*; FDR = 6.0 × 10^−10^), and T3Es (e.g., *ripX* [formerly *popA*], *ripAB*, *ripAC*) (FDR = 4.27 × 10^−4^), corresponding to the reduced growth rate, swimming motility, and HR of Δ*phcX*. Notably, although EPS-related genes were not significantly enriched as a whole set, two major regulatory genes of EPS production, namely, *epsR* (log_2_FC = 2.67) and *xpsR* (log_2_FC = 1.33), were significantly up-regulated. The overall down-regulation of the *eps* operon might be the integrated output of both regulators. On the other hand, the enrichment and overall up-regulation of adhesion-related genes (e.g., *RSp1180*, *RSp0116*; FDR = 3.2 × 10^−3^) are consistent with the enhanced biofilm formation of Δ*phcX*. As for CWDEs, two primary polygalacturonase-encoding genes, *pehB* (log_2_FC = −2.91) and *pglA* (log_2_FC = −1.84), were significantly down-regulated, which may be responsible for the reduced polygalacturonase activity. For QS-related genes, while the expression of *phcA* and the *phc* operon was not affected, *solI/solR* and *rasI/rasR*, which encode the two *luxI*/*R* homologous QS systems, and *lecM*, which is related to biofilm formation and phc-QS regulation (44), were significantly up-regulated. Additionally, our reverse transcription-PCR (RT-PCR) results suggested that *phcX* is transcriptionally independent of the *phc* operon (Fig. S3b). Other enriched gene sets include the HrpB-activated operon HDF biosynthesis genes (FDR = 2.11 × 10^−4^) (53), T2SS (FDR = 0.015), T6SS (FDR = 0.010), LPS (FDR = 0.026), and siderophore biosynthesis (FDR = 0.016) (Table S3E).

### *phcX* and *phcA* regulate multiple *phc* QS-dependent phenotypes and associated genes in opposite patterns

**FIG 5:**
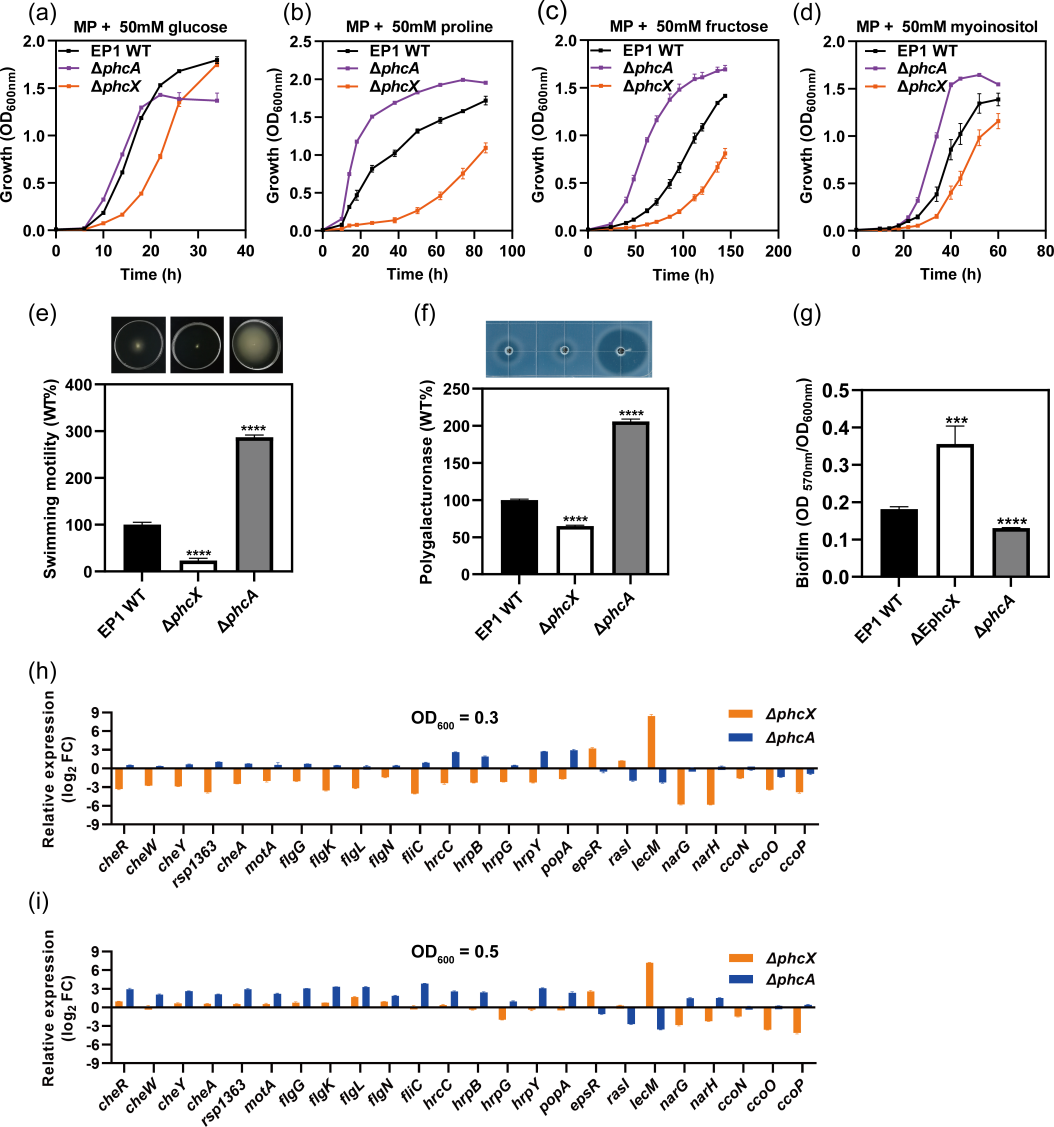
*phcX* and *phcA* regulate multiple *phc* QS-dependent phenotypes and associated genes in opposite patterns. Phenotypic characterizations of *R. solanacearum* wild-type EP1, Δ*phcX*, and *phcA* include growth curves in MP medium with 50 mM glucose (**a**), proline (**b**), fructose (**c**), or myoinositol (**d**) as the sole carbon substrate, swimming motility (**e**), polygalacturonase activity (**f**), and biofilm formation (**g**). RT-qPCR analysis of selected genes in multiple functional categories in Δ*phcA* and Δ*phcX* cultured in CTG medium until an OD_600_ of 0.3 (**h**) and an OD_600_ of 0.5 (**i**). The *recA* gene was used as an internal control for RT-qPCR. The differential expression results of mutant strains were shown in log_2_(fold change) compared with the wild-type strain. RT-qPCR assays were conducted at two cell densities to validate our RNA-seq results of Δ*phcX* (OD_600_ of 0.3) and the reported transcriptome data of Δ*phcA* (OD_600_ of 0.5), respectively. The experiments were repeated at least three times, with three technical replicates per experiment. The data were presented as mean ± standard deviation.

Our results demonstrated that growth, swimming motility, and polygalacturonase activity were positively regulated by PhcX. Interestingly, several studies have reported the negative regulation of these phenotypes by PhcA (4, 31). To further confirm this observation, we generated a *phcA* deletion mutant (Δ*phcA*) in EP1 and compared the phenotypes of the wild-type EP1, Δ*phcX*, and Δ*phcA* side by side. Our results showed that Δ*phcA* grew faster than the wild-type EP1 in minimal media with one of sucrose, glucose, fructose, myoinositol, or proline as the sole carbon substrate, while Δ*phcX* grew at slower rates than the wild-type strain under the same conditions (Fig. 5a through d). Δ*phcA* also exhibited significantly increased swimming motility and polygalacturonase activity, as well as decreased adherent biofilm formation *in vitro*, opposite to the phenotypes of Δ*phcX* (Fig. 5e through g).

Similarly, our transcriptome analysis showed that the deletion of *phcX* led to the up-regulation of swimming-related genes and the *solIR* and *rasIR* QS systems, which were reported to be positively regulated by PhcA (30). To better understand the differences in the regulatory roles of PhcX and PhcA, we compared the DEGs identified in our RNA-seq analysis of Δ*phcX* with those reported in a previous transcriptome study of Δ*phcA* in GMI1000 (30). It should be noted that, due to differences in the gene contents, we focused our comparison on the 4,390 one-to-one orthologous genes between EP1 and GMI1000 (Table S4A and B).

**FIG 6:**
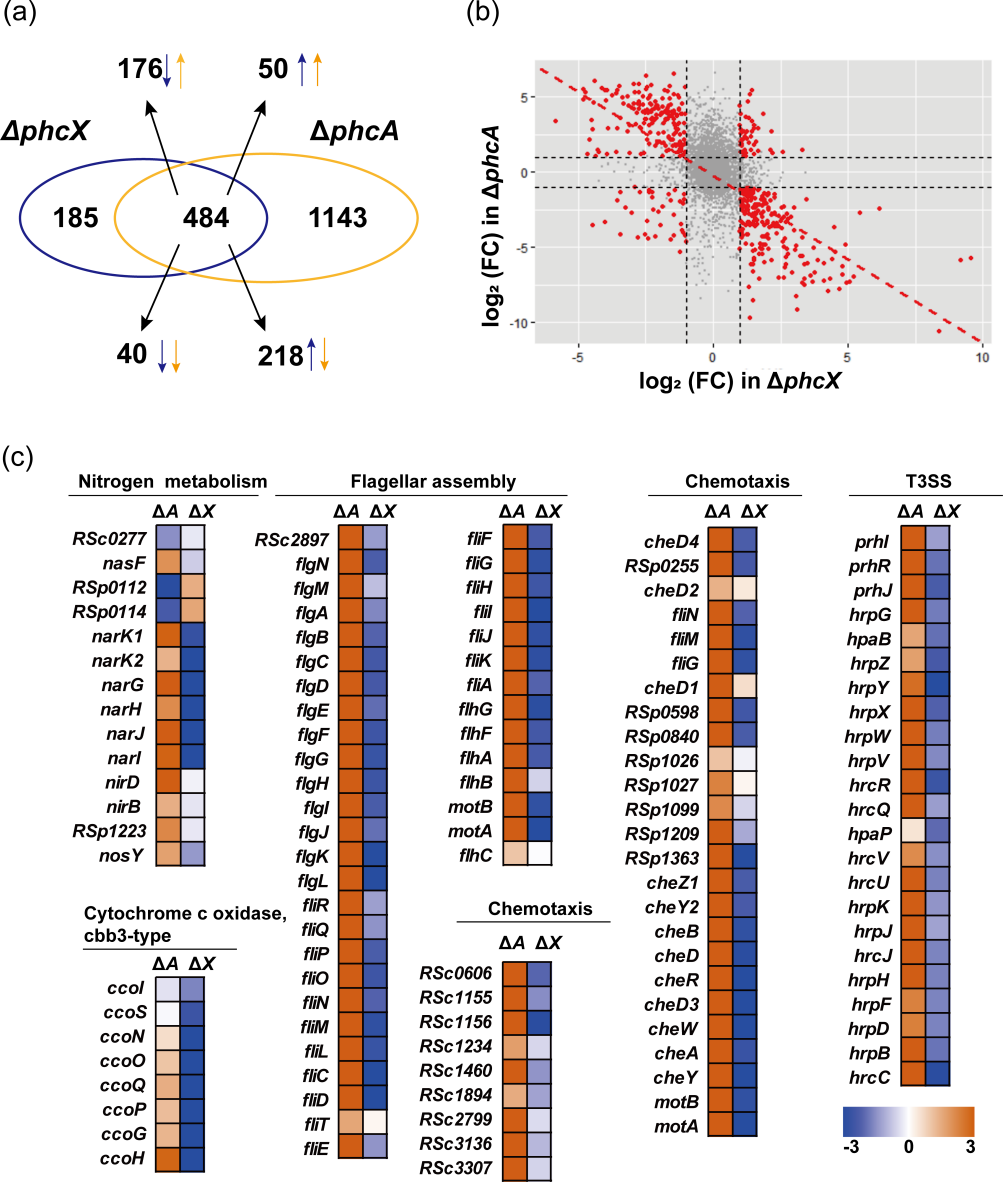
Comparison between the differential gene expression patterns of Δ*phcX* and Δ*phcA*. (**a**) The Venn diagram depicts the numbers of DEGs (|log_2_FC| ≥ 1, FDR <0.05) shared by both Δ*phcX* (blue) and Δ*phcA* (yellow) or unique to either mutant. Upward/downward arrows indicate up-/down-regulated genes, respectively. (**b**) The scatter plot shows, for all one-to-one orthologous genes between EP1 and GMI1000, the relative expression patterns of each gene in Δ*phcX* (x-axis) and Δ*phcA* (y-axis). Dots in red stand for DEGs detected in both Δ*phcX* and Δ*phcA*, and the dashed line shows the correlation (*r* = −0.70, *P* < 10^−15^). (**c**) Heat maps show, for genes in selected virulence-related functional categories, the differential expression patterns of each gene in Δ*phcX* and Δ*phcA*. The blue and yellow colors correspond to down- and upregulation, respectively.

Using the same criteria (FDR < 0.05 and |log_2_FC| ≥ 1), we identified 1,627 genes differentially expressed in Δ*phcA*, considerably more than the 699 DEGs detected in Δ*phcX*. Then, 922 and 705 of the DEGs in Δ*phcA* were up-regulated and down-regulated, respectively. Notably, 484 of the DEGs detected in Δ*phcX* (69.24%) were also differentially expressed in Δ*phcA*, among which 394 genes (81.40%) were regulated in opposite directions in Δ*phcX* and Δ*phcA* (Fig. 6a). In addition, the 484 DEGs detected in both Δ*phcX* and Δ*phcA* exhibited significantly higher degrees of differential expression than the DEGs specific to either Δ*phcX* or Δ*phcA* (Wilcoxon rank-sum test, *P* < 10^−9^) (Fig. S3c and d). We also found a significant negative correlation (*r* = −0.43, *P* < 10^−15^) between genes’ log_2_ FC values in Δ*phcX* and Δ*phcA* for all genes, and the negative correlation was relatively strong (*r* = −0.70, *P* < 10^−15^) for the DEGs detected in both mutants (Fig. 6b). In particular, the genes related to nitrate reductase, swimming motility, chemotaxis, T3SS, hdf biosynthesis, adhesion, and QS all showed an overwhelming trend of opposite differential expression patterns in Δ*phcX* and Δ*phcA* (Fig. 6c; Table S4C). This pattern revealed by transcriptome data was independently validated by RT-qPCR assays of representative genes from each category (Fig. 5h and i). Overall, our results reveal a significant overlap between the genes regulated by PhcX and PhcA and contrasting modes of regulation between the two regulators.

### Deletion of *phcX* results in similar phenotypes in *R. solanacearum* strains GMI1000 and EP1

To evaluate if the functions of *phcX* are conserved in other *R. solanacearum* strains, we generated the *phcX*-deletion mutant in *R. solanacearum* strain GMI1000 (GMI1000-Δ*phcX*) (Table S1B) and analyzed its phenotypes. Compared with the wild-type GMI1000, GMI1000-Δ*phcX* exhibited a much slower growth rate at the early stage, substantially attenuated swimming/twitching motilities and polygalacturonase activity, and significantly increased biofilm production, all consistent with the phenotypes of EP1-Δ*phcX* (Fig. S4a through f and h). At the same time, the deletion of *phcX* in GMI1000 had no significant influence on cellulase activity (Fig. S4g). Interestingly, the deletion of *phcX* led to substantially increased EPS production in GMI1000 (*P* < 0.0001), although it took twice as long for GMI1000-Δ*phcX* to reach an OD_600 nm_ of 1.0 compared with the wild-type strain (34 vs 17 h) (Fig. S4i). Transformation of native PhcX recovered the phenotypes of GMI1000-Δ*phcX* to those of the wild-type GMI1000. Our results suggest that functions of PhcX were largely conserved in these two phylotype I *R. solanacearum* strains, GMI1000 and EP1.

### Phylogenetic analysis identifies PhcX as the ortholog of LqsR

**FIG 7:**
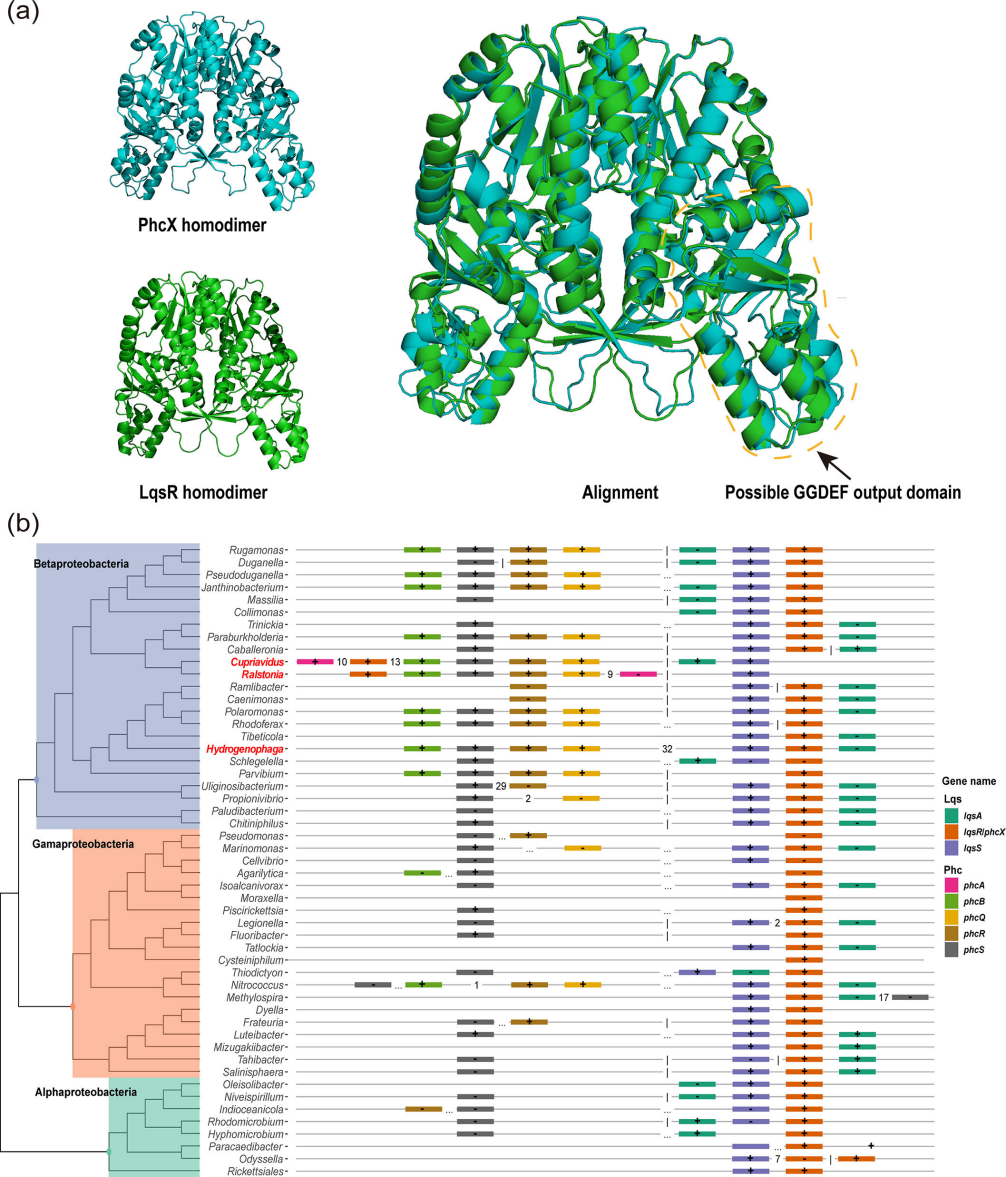
Genomic analysis suggests that PhcX likely originated as part of the Lqs QS system. (**a**) Predicted protein structures of the PhcX homodimer in *R. solanacearum* EP1, the LqsR homodimer in *L. pneumophila* C9_S, and their structural alignment. The protein structures were predicted using AlphaFold2 and visualized and aligned using PyMOL. The yellow dashed-line circle highlights the diguanylate cyclase (GGDEF) output domain of PhcX and LqsR in their homodimer structure models. (**b**) The presence/absence patterns and genomic arrangements of *phc* and *lqs* genes in Proteobacteria. The genus-level phylogeny shown on the left was extracted from Genome Taxonomy Database. For each genus, the representative genomic arrangement was shown. Each *phc* or *lqs* gene is represented by a different color. In the graphical representation of genomic arrangements, immediate neighboring genes are connected by a solid line; genes separated by a few (1 to 49) genes are connected with the number shown in between; genes separated by more than 50 genes are connected by a dotted line; and genes on different replicons (or assembled genomic sequences) are separated by a vertical line. The “+” and “−” signs in gene boxes indicate that the corresponding genes are on the forward and reverse strands, respectively. The three genera in which *phcX/lqsR* is located adjacent to the *phc* QS genes, including *Hydrogenophaga*, *Cupriavidus*, and *Ralstonia*, are highlighted in red.

To further investigate the distribution of PhcX in other bacteria, we queried the OrthoDB v11 database and found that PhcX belongs to an orthologous group (ID: 410377at1224) consisting exclusively of genes from Proteobacteria. We then retrieved all the 6,937 representative genomes (each representing a species) of Proteobacteria from the NCBI RefSeq database and compared each genome with the EP1 genome to identify potential orthologs of PhcX. In total, we found orthologs of PhcX in 310 representative genomes, including 16 Alphaproteobacteria, 206 Betaproteobacteria, 1 Deltaproteobacteria, and 87 Gammaproteobacteria (Table S5 and Fig. S5). Notably, PhcX was found to be orthologous to the LqsR of *Legionella pneumophila*, the causal agent of Legionnaires' disease (54). The orthology between PhcX and LqsR was further supported by our phylogenetic analysis of PhcX/LqsR homologs in Proteobacteria (Fig. S5). PhcX and LqsR have the same response regulator receiver domain and share a protein sequence similarity of 50.45%. In addition, the predicted structures of the two proteins were highly similar, with a root mean square deviation (RMSD) value of only 1.064 (Fig. 7a).

LqsR is the response regulator of the Lqs QS system, which also contains an autoinducer synthase, LqsA, and two sensor histidine kinases, LqsS and LqsT. The genes encoding LqsA, LqsR, and LqsS form a gene cluster in the *L. pneumophila* genome (55). The Lqs QS system is different from the Phc QS system in both their genes and the QS signal. The Phc QS system was not found in *L. pneumophila*, whereas the autoinducer synthase and sensor kinases of the Lqs QS system were all missing in RSSC, raising an intriguing question regarding the association of PhcX and the two different QS systems during evolution.

We then systematically investigated the distributions of gene clusters encoding the Phc and Lqs QS systems in Proteobacteria. Our results showed that the *lqs* gene cluster is widely distributed in Alpha-, Beta-, and Gammaproteobacteria, while the *phc* gene cluster has a more restricted distribution in Betaproteobacteria (Fig. 7b). The two QS systems coexist in many Betaproteobacteria species; in most cases, *phcX/lqsR* belongs to the *lqs* gene cluster. Interestingly, in *Cupriavidus*, *phcX/lqsR* is de-associated from *lqsA* and *lqsS* but relocated to the proximity of the *phc* gene cluster. Finally, *phcX/lqsR* becomes an immediate neighbor of the *phc* gene cluster in *Ralstonia*, where *lqsA* is lost in all its members except for *R. insidiosa*. Our phylogenetic results suggest that PhcX/LqsR was ancestrally part of the Lqs QS system and became associated with the Phc QS system gradually during the evolution of *Ralstonia*.

## DISCUSSION

In this study, we report the identification and functional characterization of *phcX*, encoding a new regulator of metabolism and virulence in *R. solanacearum*. Results from *in vitro* experiments on *R. solanacearum* strains growing in culture showed that the *phcX* deletion mutant (Δ*phcX*) displayed multiple significant phenotypic alterations, including a prolonged lag phase, substantially reduced swimming and twitching motilities, decreased polygalacturonase activity and EPS production, and increased biofilm formation. *In planta* assays further demonstrated that *phcX* deletion leads to delayed HR and impaired virulence. PhcX is predicted to be a response regulator, and the deletion of *phcX* resulted in differential expression of a wide range of genes, many of which directly correspond to the phenotypic outcomes. Although *phcX* is located immediately upstream of the *phc* operon, it is transcribed independently, and the expression of *phc* genes was not significantly different in Δ*phcX*, suggesting that the phenotypes of Δ*phcX* were caused by disruption of PhcX functions. In addition, the Δ*phcX* phenotypes were successfully restored by *phcX* complementation. These lines of evidence all support the important regulatory functions of PhcX in *R. solanacearum*.

The Δ*phcX* mutant grew much slower than the wild-type strain at the early proliferation stage (Fig. 1), and RNA-seq analysis revealed the enrichment of GO and KEGG terms on nitrogen metabolism among down-regulated genes (Table S3C and D), suggesting a crucial role of PhcX in metabolism. The metabolism and energy generation processes are required by pathogens to thrive during their infection of host tissues. PhcA and EfpR, two of the most important regulators in *R. solanacearum*, play dual roles in regulating both virulence and metabolism (31, 56). In Δ*phcX*, all *nar* genes related to the nitrate reductase pathway were significantly down-regulated, especially *narG* (log_2_FC = −5.8) (Table S4E). Several studies in *R. solanacearum* have highlighted the importance of nitrogen metabolism in root attachment, stem colonization, and virulence in the host xylem (21, 57); in particular, Δ*narG* mutant grows poorly on NO_3_^−^ and is metabolically impaired compared to the wild-type. Another set of metabolic genes dramatically down-regulated in Δ*phcX* was the cytochrome cbb3 oxidases operon (*ccoSNOQPGH*) (Table S4E), including *ccoN*, whose disruption was sufficient to abolish cbb3-type cytochrome c activity in *Vibrio cholerae* (58). In Proteobacteria, cytochrome cbb3 oxidases are required for the colonization of oxygen-limited environments and thus may be an important determinant of pathogenicity (59). For instance, cbb3-type cytochrome c oxidase was required for the full virulence and multiplication of *R. solanacearum* R3bv2 in microaerobic environments (22). These observations suggest that PhcX might control the growth of *R. solanacearum* positively in soil, rhizosphere, and plants by regulating nitrogen metabolism and cytochrome c oxidase activity.

Swimming and twitching motilities, chemotaxis, and polygalacturonase activity are all essential for plant colonization and full virulence in *R. solanacearum* at the early stages of infection, when bacterial population densities are low (60). The swimming motility mediated by flagella is activated by FlhDC, which is positively regulated by PehSR and VsrBC and negatively regulated by MotN and VsrAD (61). PehSR is also a positive regulator of twitching motility and polygalacturonase activity (62), while PhcA negatively regulates these traits by repressing PehSR (43). Interestingly, although Δ*phcX* exhibited substantially reduced motilities and polygalacturonase activity (Fig. 2a through c), as well as a broad down-regulation of flagellar and polygalacturonase genes (Table S3E), most of the regulatory genes (e.g., *flhD/C*, *pehS/R*, *vsrAB*, *vsrCD*, and *phcA*) were not differentially expressed (Table S3B). *motN*, which encodes a repressor of motility, was even down-regulated (log_2_FC = −1.7) (Table S3B). Similarly, many chemotaxis-related genes were down-regulated in Δ*phcX* but are not known to be regulated by PhcA, PehSR, etc. Therefore, PhcX likely controls motility and polygalacturonase activity via a mechanism independent of PhcA and other unknown regulators.

Biofilm is a crucial component of virulence in *R. solanacearum*, and its formation involves adhesins and lectins. We found in Δ*phcX* an overall up-regulation of adhesion-related genes, particularly *rsl* (also named *lecF*; log_2_FC = 8.4) and *lecM* (log_2_FC = 9.5) (Table S3B), two lectin-encoding genes. It has been shown in strain OE1-1 that the LecM positively regulates biofilm formation and facilitates the attachment of OE1-1 to the host cells in intercellular spaces (8). Consistent with this, both the floating pellicle at the air-liquid interface and adherent biofilm formation were significantly increased in Δ*phcX* (Fig. 2e). These findings indicate that PhcX exerts strong repressive control on biofilm formation.

EPS accumulates as *R. solanacearum* invades and multiplies *in planta* and blocks the flow of water and other nutrients, eventually leading to severe wilting symptoms. EPS production requires the *eps* gene cluster (*epsABCDEF*) and is regulated by multiple activators (e.g., XpsR, VsrAD, and VsrBC) and one repressor (e.g., EpsR). Deletion of *phcX* resulted in reduced EPS production (Fig. 2f) and a mild down-regulation of the entire *eps* gene cluster, which might be the integrated output of up-regulated expression of both *epsR* (log_2_FC = 2.67) and *xpsR* (log_2_FC = 1.33) (Table S3E and F).

After invading host intercellular spaces, *R. solanacearum* uses T3SS to secrete an arsenal of T3Es to subvert plant defense responses and promote virulence (20). It is proposed that the pathogens sense plant signals by PrhA and transduce the signals to PrhJ via PrhI/R; PrhJ affects the expression of HrpG, which positively regulates HrpB together with PrhG; HrpB directly controls the expression of T3SS and T3Es genes (39, 63). The T3SS regulatory network also contains other regulators, such as PrhN, PrhO, PrhP, and CysB (64). PhcA represses the expression of PrhI/R directly, thus negatively regulating HrpG and, in turn, T3SS/T3Es (65). In our study, Δ*phcX* exhibited delayed HR development and attenuated virulence (Fig. 3a), accompanied by substantially lower expression of PrhJ/HrpG/HrpB (Table S3A and E) and a global down-regulation of T3SS and T3Es, as well as the HrpB-activated operon of six HDF genes responsible for the synthesis of a tryptophan derivative (Table S3E). Overall, these results suggest that PhcX plays a critical role in the plant-pathogen interaction and may activate the expression of T3SS/T3E genes via both PrhJ- and HrpG-mediated pathways.

**FIG 8:**
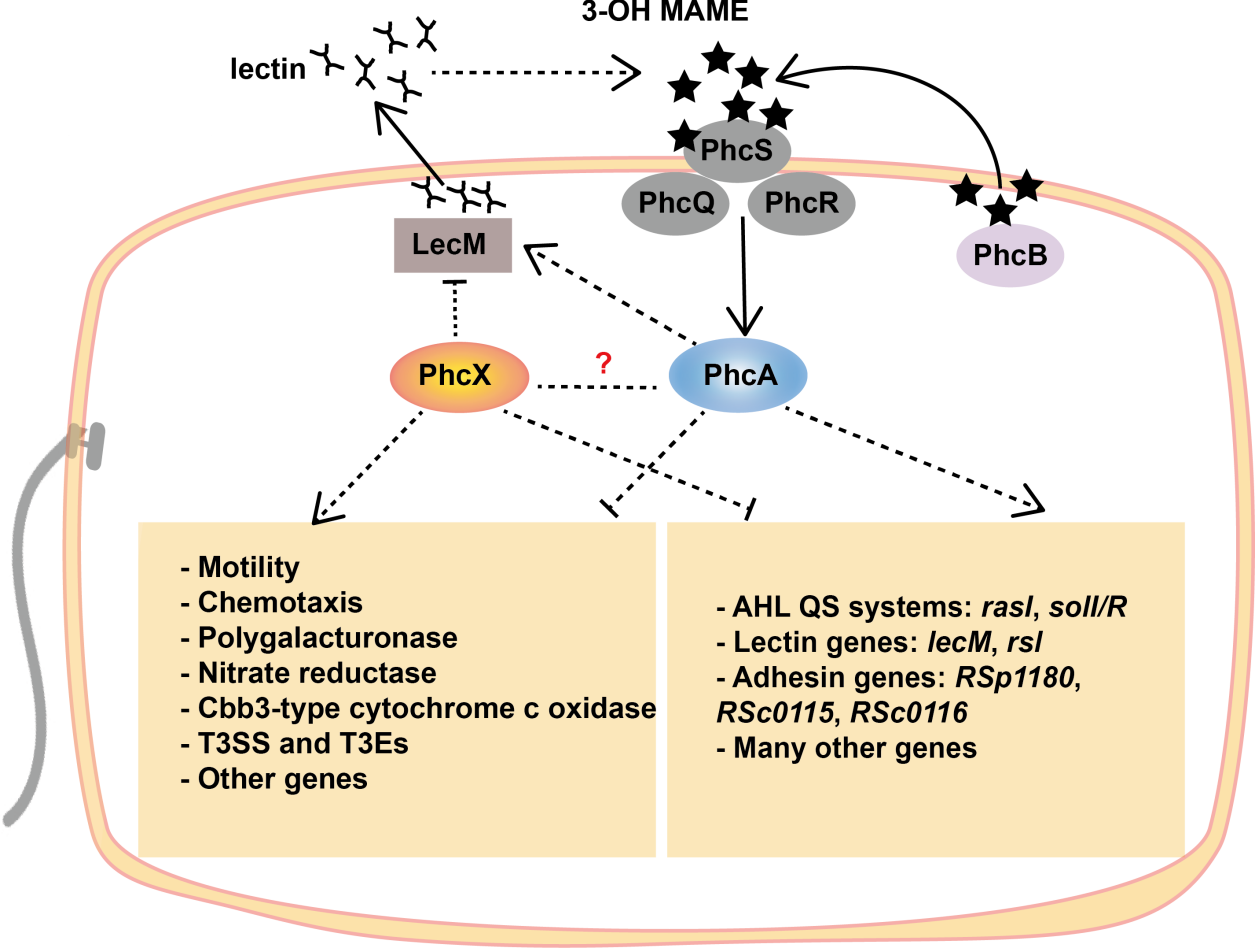
Schematic representation of the PhcX and PhcA regulatory networks in *R. solanacearum* EP1. Many genes associated with virulence (e.g., genes related to motility, chemotaxis, and genes encoding polygalacturonases, T3SS and T3Es, lectins, and adhesins), metabolism (e.g., genes encoding nitrate reductases and cbb3-type cytochrome c oxidases), and regulation (e.g., genes in AHL QS systems) are regulated by both PhcX and the global virulence regulator PhcA, but in opposite directions. PhcX might interact indirectly with the phc QS system through the down-regulation of *lecM*, which positively affects the stability of 3-OH MAME, the phc QS signal. It remains to be investigated if other indirect or direct regulations exist between PhcX and PhcA. Dashed lines indicate potential indirect regulation. Lines ending with an arrowhead indicate positive regulation, while lines ending with a crossbar indicate negative regulation.

Our results also provide insights into the relationships between PhcX and other regulatory mechanisms of *R. solanacearum* (Fig. 8). Several gene regulation-related GO terms (molecular transducer activity and signaling) and KEGG pathways (two-component system), as well as the gene set on QS, were enriched among genes differentially expressed in Δ*phcX*. Importantly, our transcriptome analyses revealed a significant overlap between the regulatory ranges of PhcX and the master regulator PhcA, including many of the abovementioned genes associated with Δ*phcX* phenotypes. Furthermore, PhcX and PhcA showed contrasting regulatory modes on most shared targets, and their mutants exhibited phenotypic alterations in opposite directions (Fig. 5 and 6). For instance, genes in the *solIR* and *rasIR* QS systems were downstream of both regulators but were repressed by PhcX and activated by PhcA (Table S4C). Intriguingly, the expression of *phcA* and phc QS genes was not affected in Δ*phcX* (Table S4B), suggesting that PhcX and PhcA may execute their regulatory functions independently, or one may regulate the other at post-transcription levels. In particular, it has been proposed that the production of active PhcA is post-transcriptionally regulated by PhcR (25). The regulatory relationship between PhcX and PhcA remains to be further investigated, for instance, by examining the protein abundance of PhcA (or PhcX) in Δ*phcX* (or Δ*phcA*).

Interestingly, our RT-qPCR results (Fig. 5h and i) showed that the regulatory effects of PhcX were more evident at an OD_600_ of 0.3 than at an OD_600_ of 0.5. Given that Δ*phcX* exhibited a prolonged lag phase and the traits positively regulated by PhcX are generally important for the early stages of infection, it is possible that PhcX might function early on during the transition from survival and early invasion mode to high virulence status. It remains to be further investigated if PhcX acts after PhcA or responds to other QS signals. On the other hand, although the phc QS pathway is normally activated at a much lower cell density (OD_600_ <0.1), the |log_2_FC| values of most examined genes were greater at an OD_600_ of 0.5 than at an OD_600_ of 0.3 in Δ*phcA*. This pattern reflects the dynamic nature of gene regulation in *R. solanacearum* and suggests that the regulatory functions of genes like *phcX* and *phcA* might need to be investigated at multiple cell densities from low to high to achieve a more complete understanding.

Although *phcX* was first discovered and investigated in *R. solanacearum* EP1, we also showed in GMI1000 that the deletion of *phcX* led to largely similar phenotypes as in EP1 (Fig. S4). In addition, our genomic analysis revealed that *phcX* is present and clustered with the *phcBSRQ* operon in nearly all *Ralstonia* genomes and PhcX is highly conserved at the protein sequence level (average identity >96%) (Table S2). These lines of evidence all support the idea that the functions of *phcX* might be relatively conserved in RSSC.

Outside RSSC, *phcX* is also broadly present in more than 300 Proteobacteria genomes (Table S5; Fig. S5). Notably, we found that *phcX* was orthologous to *lqsR*, encoding the response regulator LqsR of the Lqs QS system in *L. pneumophila*, the causal agent of Legionnaires’ disease. Lqs QS system plays important regulatory roles in *L. pneumophila* to modulate its life cycle, pathogen-host cell interactions, motility, and natural competence (55). Besides LqsR, the Lqs system also consists of the pyridoxal-5-phosphate-dependent autoinducer synthase LqsA and two homologous membrane-bound sensor histidine kinases LqsS and LqsT (66). The Lqs and Phc QS systems have completely different signals and genes, and they coexist in numerous Betaproteobacteria genomes, including *Cupriavidus* spp. and *Ralstonia insidiosa*, which are closely related to RSSC. Interestingly, *phcX*/*lqsR* is clustered with *lqsA* and *lqsS* in most cases but becomes disassociated with *lqs* and linked to the *phc* gene cluster instead in *Cupriavidus* spp. and *R. insidiosa*, and *lqsA* is further lost in RSSC and most other *Ralstonia* (Fig. 7b). It was shown recently in *Cupriavidus taiwanensis* that both Lqs and Phc are functional (67); however, the Lqs system seems to have few influences on growth rate, swimming motility, biofilm formation, or EPS production, unlike the Phc system in *C. taiwanensis* or the *R. solanacearum* PhcX examined in this study. Altogether, the evolutionary dynamics of Phc and Lqs as well as the functional characterization of PhcX suggest that, during the evolution of *Ralstonia*, PhcX might have experienced network rewiring and acquired new regulatory roles along with the breakdown of the Lqs system.

Strikingly, the predicted structures of PhcX and LqsR are almost identical (Fig. 7a), indicating highly similar molecular functions. A recent structural analysis of monomeric LqsR suggested that the putative output domain of LqsR forms a five-stranded antiparallel β-sheet fold, similar to the diguanylate cyclase domain of PleD. LqsR also has a degenerate form of the conserved GG[DE][DE]F sequence pattern, which is a signature of diguanylate cyclases, suggesting that the output function of LqsR might be related to c-di-GMP metabolism and the signaling network (54). However, the study did not detect any diguanylate cyclase activity of LqsR *in vitro*. Therefore, it awaits further investigation to elucidate the regulatory mechanisms of PhcX and LqsR.

## MATERIALS AND METHODS

### Bacterial strains, plasmids, and growth conditions

The bacterial strains and plasmids used in this study are presented in Table S1B. All *R. solanacearum* strains were routinely grown in CTG-rich medium (casein acid hydrolysate 1 g/L, tryptone 10 g/L, glucose 5 g/L) (68) at 28°C. CTG medium was supplemented with 0.005% 2,3,5-triphenyltetrazolium chloride (final concentration) in agar plates. *Escherichia coli* strains were grown in Luria–Bertani medium at 37°C. Antibiotics were added at the following final concentrations (µg/mL) when necessary: kanamycin (50), gentamicin (50), and rifampicin (25). The competent cells of *R. solanacearum* strains for homologous recombination were generated by growing in MM broth with 10% glycerol. Bacterial growth was determined by measuring the optical density at a wavelength of 600 nm.

### *In vitro* passage experiment and detection of mutation

For each passage, a single colony of the EP1 strain was grown overnight in a liquid CTG medium at 28℃. One hundred microliters of the suspension was transferred onto a CTG agar plate and spread uniformly. The plate was inoculated for 48 h at 28℃. Freshly grown bacterial populations were then washed down with 1 mL sterile water. Next, 100 µL of the suspension was spotted onto a new CTG agar plate for the next round as described above, and another 100 µL was used for serial dilutions to detect EPS-deficient mutants. Dark red granular colonies with reduced EPS production were chosen as EPS-deficient mutants and further confirmed on new CTG agar plates. To detect the mutation(s) causing the EPS-deficiency, genomic DNA of the ancestral wild-type strain and mutants was extracted using the EasyPure Bacteria Genomic DNA Kit following the manufacturer’s instructions, and genome sequencing was conducted on the HiSeq X Ten system to generate 150 bp pair-end reads at more than 200× depth. Delly v0.8.5, SAMtools v1.1, and BCFtools v1.10.2 were used to detect structural variants, copy number variations, and single nucleotide variations, respectively (69).

### Generation of in-frame deletion mutants and complementation

The *phcX* and *phcA* deletion mutants were generated from *R. solanacearum* EP1 and GMI1000 following the methods described previously (70). Briefly, the upstream and downstream flanking regions of their target genes and the gentamicin resistance gene sequence were PCR amplified from the *R. solanacearum* genome and plasmid pK18*mobsac*B, respectively. Then, these three fragments were conjugated with joint PCR, in which the gentamicin resistance gene sequence was added between the upstream and downstream flanking fragments by using the overlap primers. DNA fragments were mixed with the corresponding competent wild-type *R. solanacearum* strains and plated on CTG medium agar plates supplemented with gentamicin for mutant selection. The transformants were confirmed by PCR using primers and DNA sequencing. Genome resequencing was conducted for further verification to ensure that the target gene was deleted without introducing any other mutations in the genome. For complementation analysis, the native promoter sequence and coding regions of target genes were amplified and cloned into the *Hin*dIII (5′ end) and *Eco*RI (3′ end) restriction sites available in the multiple cloning region of the pBBR1-MCS2 plasmid. The *Hin*dIII restriction site is 92 nucleotides upstream of the start codon of the *lacZ* gene. The complementary plasmid was electroporated into the corresponding competent mutants. Primers used for gene deletion and complementation are listed in Table S1.

### Bacterial growth analysis under different nutrition conditions

*R. solanacearum* was cultured in CTG medium at 28°C until it reached the logarithmic growth phase. For growth analysis in CTG medium, the bacterial suspension was diluted to an OD_600 nm_ of 0.1 with fresh CPG medium and then inoculated into new CTG medium with a ratio of 1:100. For growth analysis in ground and filtered tomato stems or MP medium (39), the bacterial suspension was washed twice with sterilized water. Bacterial cells were harvested by centrifugation at 4,000 rpm for 10 min at 4°C. Then, the bacterial pellet was resuspended into ground and filtered tomato stems or MP medium containing 50 mM sole carbon source (one of glutamine, glucose, proline, fructose, or myoinositol) and adjusted to an OD_600 nm_ of 0.01. Ground and filtered tomato stems were obtained by grinding the fresh tomato stem and filtering through a 0.25-µm membrane after centrifugation at 15,000 rpm for 2 min. The growth curves were recorded using the Bioscreen-C automated growth curve analysis system or detected manually by UV-visible spectroscopy at periodic intervals. The corresponding media without bacteria were used as negative controls. The exponential growth rate and lag phase duration were determined using the R package growthrates v0.8.4 (https://github.com/tpetzoldt/growthrates). Three independent experiments were performed with at least five replicates each time.

### Swimming and twitching motility

Swimming motility was assessed on a semisolid medium plate with 0.3% agar. Then, 1 µL of bacterial suspension was stabbed into the center of plates containing 0.5% tryptone, 0.3% yeast extract, and 0.3% agar. Photos were taken, and the swimming-area diameters were measured after 24 h of incubation at 28°C. For twitching motility, it was performed as previously reported with some modifications (10). In brief, 2 µL of bacterial suspension adjusted to an OD_600_ of 0.1 was inoculated onto the surface of CTG plates with 1.6% agar. Colonies were examined, and photos with four times magnification were taken under a Leica stereomicroscope system at 24 h post-inoculation. Three independent experiments were performed with at least three replicates each time.

### Cellulase activity and polygalacturonase activity

Cellulase activity and polygalacturonase activity were tested in cellulase detection medium (1 g carboxymethyl ethyl cellulose, 3.8 g Na_3_PO_4_, 8 g agarose, pH 7.0, per liter) and polygalacturonase detection medium [5.0 g polygalacturonic acid, 2.0 g sucrose, 2.0 g (NH_4_)_2_SO_4_, pH 5.5, per liter], respectively. The bacterial culture was incubated to an OD_600_ of approximately 1.0, and 20 µL of the bacterial suspension was inoculated into the detection plates. The plates were incubated at 28°C for 48 h. The cellulase detection plates were stained with 0.5% Congo red for 30 min and then washed three times with 1 M NaCl. The polygalacturonase detection plates were stained with 1 M HCl for 15 min and then washed with 1 M NaCl. Then, the diameter of the transparent ring was measured, and photos were taken. Three independent experiments were performed with at least three replicates each time.

### Biofilm formation

The biofilm formation assay was performed in 24-well polystyrene plates as reported previously, with some modifications (8). In brief, bacterial suspensions were first adjusted to an OD_600_ of 0.1 with fresh CTG medium and then inoculated 1:20 with new CTG medium in 24-well polystyrene plates. The samples were statically incubated at 28°C for 36 h. The suspension was first mixed well, measured at OD_600_, and carefully removed. Then, 1.5 mL of 0.1% crystal violet was added to each well, and the wells were stained for 30 min. The crystal violet-stained bound cells were repeatedly rinsed with water three times and then dissolved in 95% ethanol. The absorbance at 570 nm of the solution was measured and normalized with OD_600_ to quantify biofilm formation. Three independent experiments were performed with six replicates each time.

### EPS production

EPS production in culture broth supernatant of *R. solanacearum* cells was determined by the Elson-Morgan method as described by Peyraud et al. (52). *R. solanacearum* was cultured in CTG medium at 28°C until it reached the logarithmic growth phase. The bacterial suspension was washed twice with MP medium, inoculated at an OD_600_ of 0.01, and cultured in MP minimal medium supplemented with L-glutamine (20 mM) until an OD_600_ of 1.0 at 28°C. The culture broth was then filtered with a 0.22-µm filter, and the supernatant was collected in a 2.0-mL microcentrifuge. Then, 0.4 mL of supernatant was mixed with 0.008 mL of 5 M NaCl solution and fourfold volumes of acetone. The mixture was stored at 4°C for 12 h to precipitate exopolysaccharide. Precipitated EPS pellets were dissolved in 0.4 mL H_2_O and heated at 65°C on a heat block for 10 min. Then, 0.15 mL concentrated HCl (37%) and 0.05 mL H_2_O were added. The tubes were closed tightly by adding bridges and mixed with a vortex. Tubes were transferred to the dry bath heater and maintained in the dry bath heater at 115°C for 30 min. Samples were cooled to room temperature. Then, 0.4 mL of 2 M Na_2_CO_3_ was added and homogenized, followed by 0.5 mL of 2% acetyl acetone in 1.5 M Na_2_CO_3_. The samples were then heated for 20 min with a heat block at 100°C. The samples were cooled to room temperature and transferred into 10 mL tubes. Then, 1.0 mL of 99.8% ethanol was then added and mixed well. In addition, 0.5 mL of Erlich’s reagent solution was added slowly, and the tubes were left to stand for about 30 min in the dark. The optical density of the solution was measured at 530 nm. Quantification of hexosamine was carried out by running a standard curve of N-acetyl-galactosamine that followed the overall process except precipitation.

### HR assay

The HR assay was performed on tobacco leaves with leaf infiltration. The bacterial suspension cultured in CTG medium was washed twice and adjusted to an OD_600_ of 0.1 with sterilized water. Then, 10 µL of bacterial suspension was inoculated into leaves with blunt syringes, and the development of necrotic lesions was recorded periodically.

### Virulence assays

About 6-week-old tomato plants of cultivar Zhongshusihao were inoculated with *R. solanacearum* strains according to stem injection and root dipping methods. The bacterial suspension cultured in CTG medium was washed twice and adjusted to an OD_600_ of 0.1 with sterilized water. Toothpicks were used to create a wound on each tomato stem about 3 cm above the root. Then, 2 µL of bacterial suspension was inoculated into the wound. Sterilized water without bacteria was used as the negative control. For the root dipping method, tomato seedlings were soaked in bacterial suspensions (OD_600_ of 1.0) for 30 min and then exposed to air for 15 min on dry paper. Inoculated plants were grown in a plant incubator with a 12-h light and 12-h dark cycle at 28°C with 80% relative humidity. The number of dead plants was recorded daily.

### RT-qPCR and RT-PCR

Total RNA was extracted from *R. solanacearum* strains grown in CTG medium of specific OD_600_ by the use of the Eastep Super Total RNA Extraction Kit (Promega). Thereafter, cDNA synthesis and RT-qPCR analysis were performed with the ChamQ Universal SYBR qPCR Master Mix (Vazyme) on the Applied Biosystems QuantStudio 6 Flex Real-Time PCR System (Thermo Fisher Scientific) following the manufacturer’s instructions. *recA* was used as an internal standard for each cDNA sample, and the relative level of gene expression was calculated using the ΔΔCt method. Primers used for RT-qPCR analysis were designed by the online tool PrimerQuest (https://sg.idtdna.com/PrimerQuest/Home/Index) and listed in Table S1. As for RT-PCR, once the total RNA was extracted, DNA contaminant elimination and cDNA synthesis were performed following the instructions (*TransScript* One-Step gDNA Removal and cDNA Synthesis SuperMix). PCRs were performed using standard procedures with an RNA sample without reverse transcriptase, genomic DNA, and cDNA as templates. Primers used for RT-PCR were listed in Table S1C.

### RNA-seq analysis

Total RNA was extracted from the wild-type EP1 and the *phcX* deletion mutant strains growing in CTG medium with a comparable OD_600_ of 0.3 using the Eastep Super Total RNA Extraction Kit (Promega). For each strain, three biological replicates were analyzed. Genomic DNA contamination was removed using RNasefree DNase I (Transgen) according to the manufacturer’s instructions. Total RNA was sent to Novogene (Beijing, China) for sequencing on the Illumina (CA, USA) NovaSeq 6000 platform. The raw reads were quality filtered with fastp v0.21.0 (71), and the resulting clean reads were mapped to the reference genome with bowtie2 v2.3.5.1 (72). FeatureCounts v2.0.2 was used to quantify the gene expression level (73). PCA and differential expression analysis were carried out with DESeq2 v1.28.1 (74). Genes with |log_2_FC| ≥1 and FDR <0.05 were considered significantly differentially expressed. For functional annotation, each protein sequence of EP1 was searched against the Uniport database using BLASTP v2.9.0+, and the GO annotations of close homologs (identity >80% and qcovhsp >80%) were transferred to the query sequence. KEGG annotation was performed by eggnog-mapper v2.1.4–2 (75). ClusterProfiler was used to perform gene over-representation analysis and GSEA (76).

### Bioinformatic and genomic analyses of PhcX

The gene annotations in GFF format and annotated proteomes in FASTA format of 660 genomes in *Ralstonia* and 6,937 representative genomes in Pseudomonadota were downloaded from the NCBI RefSeq database (accessed on 13 February 2023). For each genome, the annotated proteome was compared with that of EP1 using InParanoid v5.0 (77) to identify the orthologs of PhcX (WP_011002641.1), PhcB (WP_011002642.1), PhcS (WP_072633512.1), PhcR (WP_011002644.1), PhcQ (WP_011002645.1), and PhcA (WP_016723740.1). In addition, each genome was compared with *L. pneumophila* C9_S using InParanoid to identify the orthologs of LqsA (WP_010948429.1), LqsR (WP_010948430.1), and LqsS (WP_010948432.1). The genomic locations of all orthologs identified in a genome were retrieved from the corresponding GFF file to determine their clustering status. For phylogenetic analysis of PhcX/LqsR, orthologous groups were constructed from protein sequences of the 310 genomes containing PhcX/LqsR homologs using OrthoFinder v2.5.4 (78). A multiple sequence alignment of the 386 proteins in the orthogroup containing PhcX/LqsR was generated using MAFFT v7.490 (79) and filtered using trimAl v1.4 (80). Phylogenetic inference was conducted using IQ-TREE v2.1.2 (81), and branch support was evaluated using the approximate Bayes test. The protein structures of homodimers of PhcX in *R. solanacearum* EP1 or LqsR in *L. pneumophila* C9_S were predicted using AlphaFold v2.3.0 (82), visualized, aligned, and calculated for RMSD value using PyMOL v2.5.2 (https://pymol.org/).

### Statistical analysis

Statistical analyses were performed with Prism 9 software (GraphPad). Results are displayed as the mean ± standard deviation. An unpaired Student’s *t*-test was carried out to compare the significance between *R. solanacearum* strains, and the *P*-value was used to indicate the statistical significance of the differences. * indicates *P* < 0.05, ** indicates *P* < 0.01, *** indicates *P* < 0.001, and **** indicates *P* < 0.0001.

## Data Availability

The raw data from our transcriptome analyses were deposited in the NCBI Sequence Read Archive under accession number PRJNA962249.

## SUPPLEMENTAL MATERIAL

Figure S1 mbio.02028-23-s0001.pdf The identification of *phcX*.

Figure S2 mbio.02028-23-s0002.pdf Estimation of growth rate parameters for wild-type EP1, Δ*phcX*, and *phcX-comp*.

Figure S3 mbio.02028-23-s0003.pdf Supplemental results of transcription analyses related to *phcXBRSQ*.

Figure S4 mbio.02028-23-s0004.pdf Phenotypic characterizations of *R. solanacearum* GMI1000 wild-type, Δ*phcX*, and *phcX-comp*.

Figure S5 mbio.02028-23-s0005.pdf Phylogenetic analysis of PhcX, LqsR, and their homologs in representative Proteobacteria genomes.

Table S1 mbio.02028-23-s0006.docx Mutations in EPS-deficient mutants; strains, plasmids, and PCR primers.

Table S2 mbio.02028-23-s0007.xlsx Genomic distribution of *phcX*, *phcA*, and *phcBSRQ* genes in 660 *Ralstonia* genomes.

Table S3 mbio.02028-23-s0008.xlsx Average log2(fold change) values of representative genes, RNAseq data, GO term enrichment analysis, and KEGG analysis.

Table S4 mbio.02028-23-s0009.xlsx Merged RNAseq data.

Table S5 mbio.02028-23-s0010.xlsx Distribution of *phc* and *lqs* genes in Alpha-, Beta-, and Gamma-proteobacteria.

ASM does not own the copyrights to Supplemental Material that may be linked to, or accessed through, an article. The authors have granted ASM a non-exclusive, world-wide license to publish the Supplemental Material files. Please contact the corresponding author directly for reuse.
