# Supplementary material for: PhcX Is a LqsR-family response regulator that contributes to Ralstonia solanacearum virulence and regulates multiple virulence factors
Source: mBio. 2023 Oct 3;14(5):e02028-23. doi: 10.1128/mbio.02028-23 (PMC10653808; doi:10.1128/mbio.02028-23)
Supplement: Figure S1 — The identification of phcX. [file mbio.02028-23-s0001.pdf]

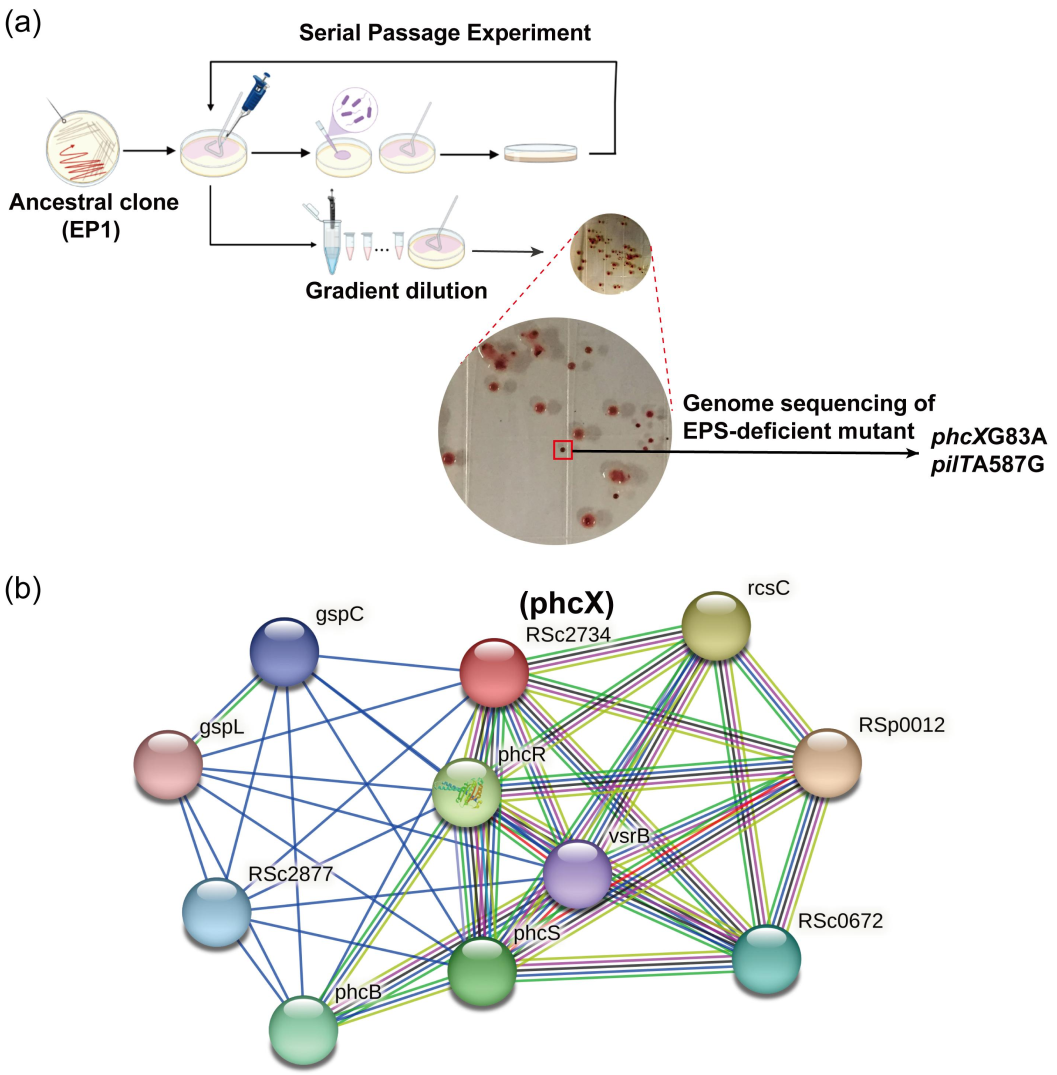


**FIG S1. The identification of *phcX*.** (a) The design and result of the *in vitro* continuous passage experiment to screen for EPS-deficient mutants. *R. solanacearum* EP1 strain was continuously cultured and detected for EPS-deficient mutants on agar plates supplemented with 0.005% 2,3,5-triphenyltetrazolium chloride (final concentration). Two mutants identified at the 23rd generation both contain a nonsynonymous mutation in the *pilT* gene (A587G) and a nonsynonymous mutation in the *phcX* gene (G83A). (b) Protein-Protein associations retrieved from the STRING database with PhcX (RSc2734) as query protein, indicating that the PhcX is strongly associated with the Phc QS system and other virulence regulators.
