# Supplementary material for: PhcX Is a LqsR-family response regulator that contributes to Ralstonia solanacearum virulence and regulates multiple virulence factors
Source: mBio. 2023 Oct 3;14(5):e02028-23. doi: 10.1128/mbio.02028-23 (PMC10653808; doi:10.1128/mbio.02028-23)
Supplement: Figure S2 — Estimation of growth rate parameters for wild-type EP1, ΔphcX, and phcX-comp. [file mbio.02028-23-s0002.pdf]

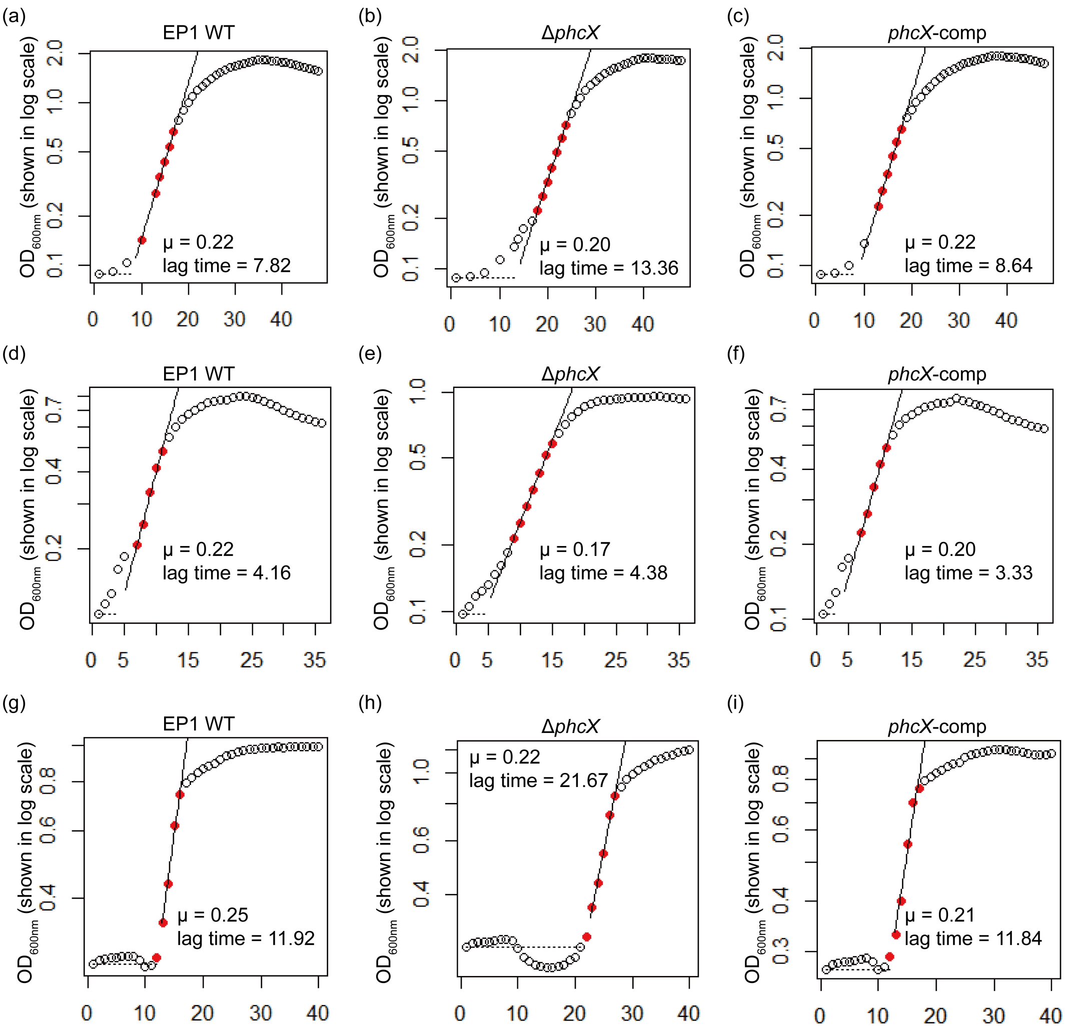

FIG S2 Estimation of growth rate parameters for wild-type EP1, Δ*phcX*, and *phcX*-comp in CTG medium (a, b, c), MP medium supplemented with 50 mM glutamine (d, e, f), and ground and filtered tomato stems (g, h, i). The exponential growth rate (μ) and lag-phase duration were estimated using the R package growth rates v0.8.4 (https://github.com/tpetzoldt/growthrates). For each graph, the X-axis shows the time in hours, and the Y-axis shows OD_{600nm} in log scale. The points in red that fall along the straight line represent the exponential growth phase, and dotted-lines indicate the lag phase.
