# Supplementary material for: PhcX Is a LqsR-family response regulator that contributes to Ralstonia solanacearum virulence and regulates multiple virulence factors
Source: mBio. 2023 Oct 3;14(5):e02028-23. doi: 10.1128/mbio.02028-23 (PMC10653808; doi:10.1128/mbio.02028-23)
Supplement: Figure S3 — Supplemental results of transcription analyses related to phcXBRSQ. [file mbio.02028-23-s0003.pdf]

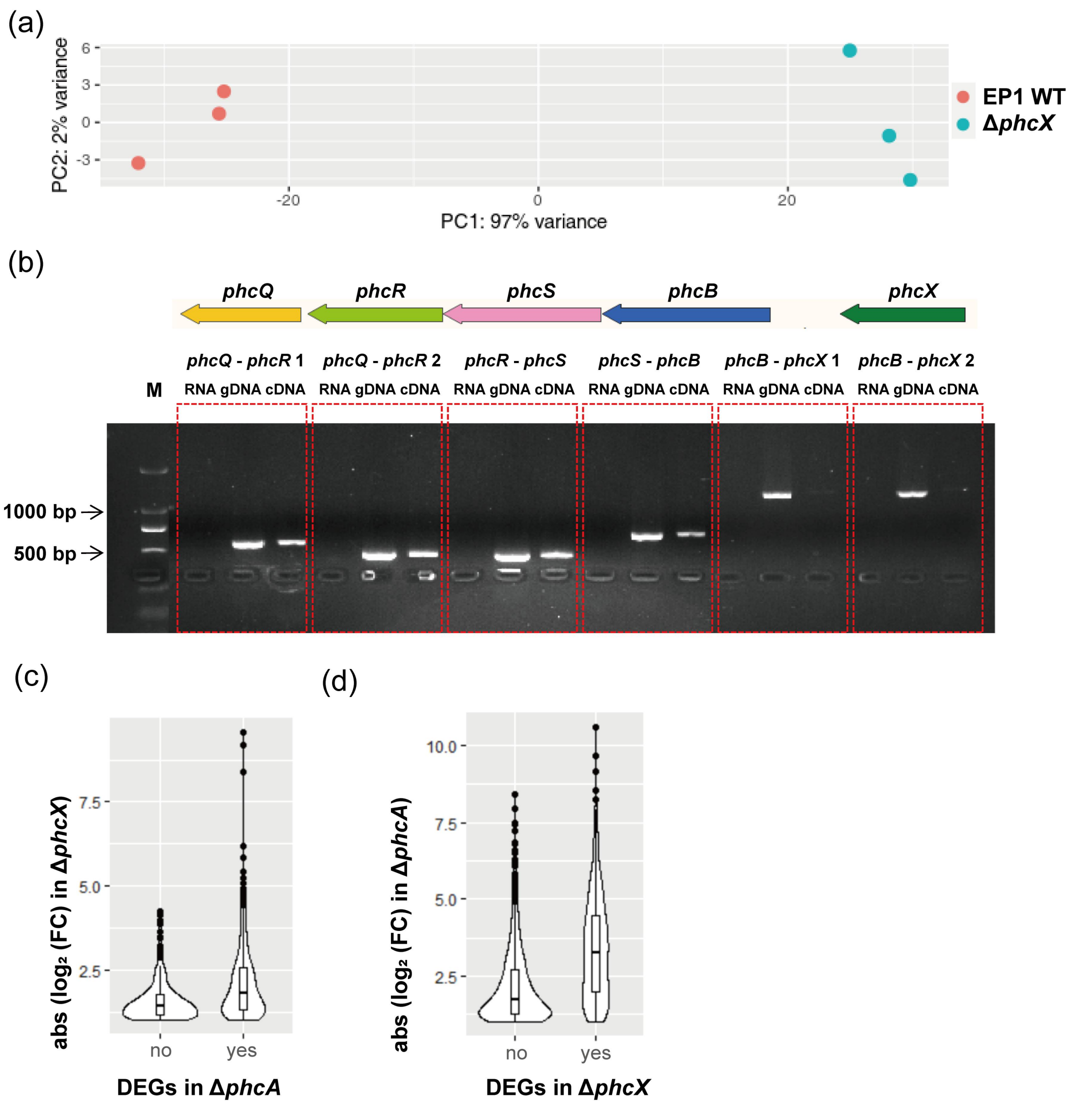

FIG S3 Supplementary results of transcription analyses related to *phcXBRSQ*. PCA analysis of the six RNA-seq samples (a). Co-transcription analysis of *phcXBSRQ* by RT-PCR (b). Each detection primer pair of two adjacent genes crossing the intergenic region was used to prepare a PCR mixture with RNA, DNA and gDNA (genomic DNA), from the EP1 strain as a template. *phcRQ* and *phcXB* were both detected by two different pairs of primer. Trans2K®Plus DNA Marker was used as the DNA marker (M). Comparison between the differential expression levels ($|\log_2 FC|$) of shared and unique DEGs detected in Δ*phcA* (c) or Δ*phcX* (d). The violin plot on the left ("no") shows the $|\log_2 FC|$ values of DEGs unique to Δ*phcA* (or Δ*phcX*), while the plot on the right ("yes") shows the $|\log_2 FC|$ values of DEGs detected in both mutants. The black rectangle in each violin plot shows the 95% CI (Confidence interval), and the black horizontal line within each rectangle shows the mean value. The $|\log_2 FC|$ values of DEGs detected in both mutants are significantly higher than those specific to either Δ*phcX* or Δ*phcA* (Wilcoxon rank-sum test, p-values $< 10^{-9}$).
