# Supplementary material for: PhcX Is a LqsR-family response regulator that contributes to Ralstonia solanacearum virulence and regulates multiple virulence factors
Source: mBio. 2023 Oct 3;14(5):e02028-23. doi: 10.1128/mbio.02028-23 (PMC10653808; doi:10.1128/mbio.02028-23)
Supplement: Figure S4 — Phenotypic characterizations of R. solanacearum GMI1000 wild-type, ΔphcX, and phcX-comp. [file mbio.02028-23-s0004.pdf]

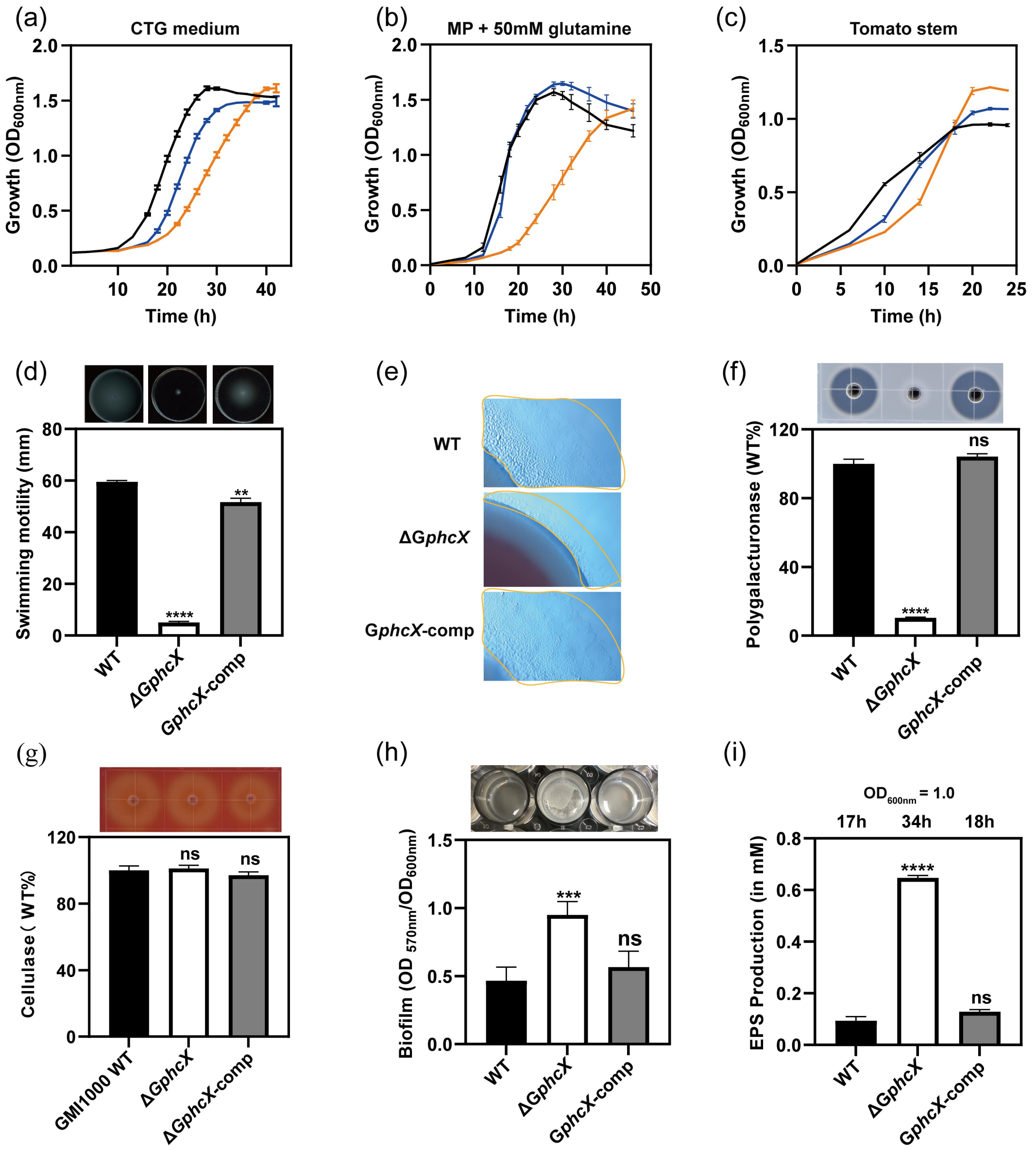

FIG S4 Phenotypic characterizations of *R. solanacearum* GMI1000 wild-type, Δ*phcX*, and *phcX*-comp. The phenotypes examined include growth curves grown in CTG medium (a), MP medium supplemented with 50 mM glutamine (b), and ground and filtered tomato stems (c), swimming motility (d), twitching motility (e), polygalacturonase activity (f), cellulase activity (g), biofilm formation (h), and EPS production (i). Results of EPS production were shown in the concentration of N-acetyl-galactosamine in mM. The numbers (in hour) on top of the panel (f) corresponded to indicate the time it took for each strain to reach OD_{600nm} of 1.0 from an initial OD_{600nm} of 0.01. All experiments were repeated three times with at least three technical replicates per experiment. The data were presented as mean ± standard deviation. Asterisks indicate values significantly different from those of wild-type EP1 (ns: no significance, * $p < 0.05$, ** $p < 0.01$, **** $p < 0.0001$, unpaired t-test).
