# Supplementary material for: PhcX Is a LqsR-family response regulator that contributes to Ralstonia solanacearum virulence and regulates multiple virulence factors
Source: mBio. 2023 Oct 3;14(5):e02028-23. doi: 10.1128/mbio.02028-23 (PMC10653808; doi:10.1128/mbio.02028-23)
Supplement: Figure S5 — Phylogenetic analysis of PhcX, LqsR, and their homologs in representative Proteobacteria genomes. [file mbio.02028-23-s0005.pdf]

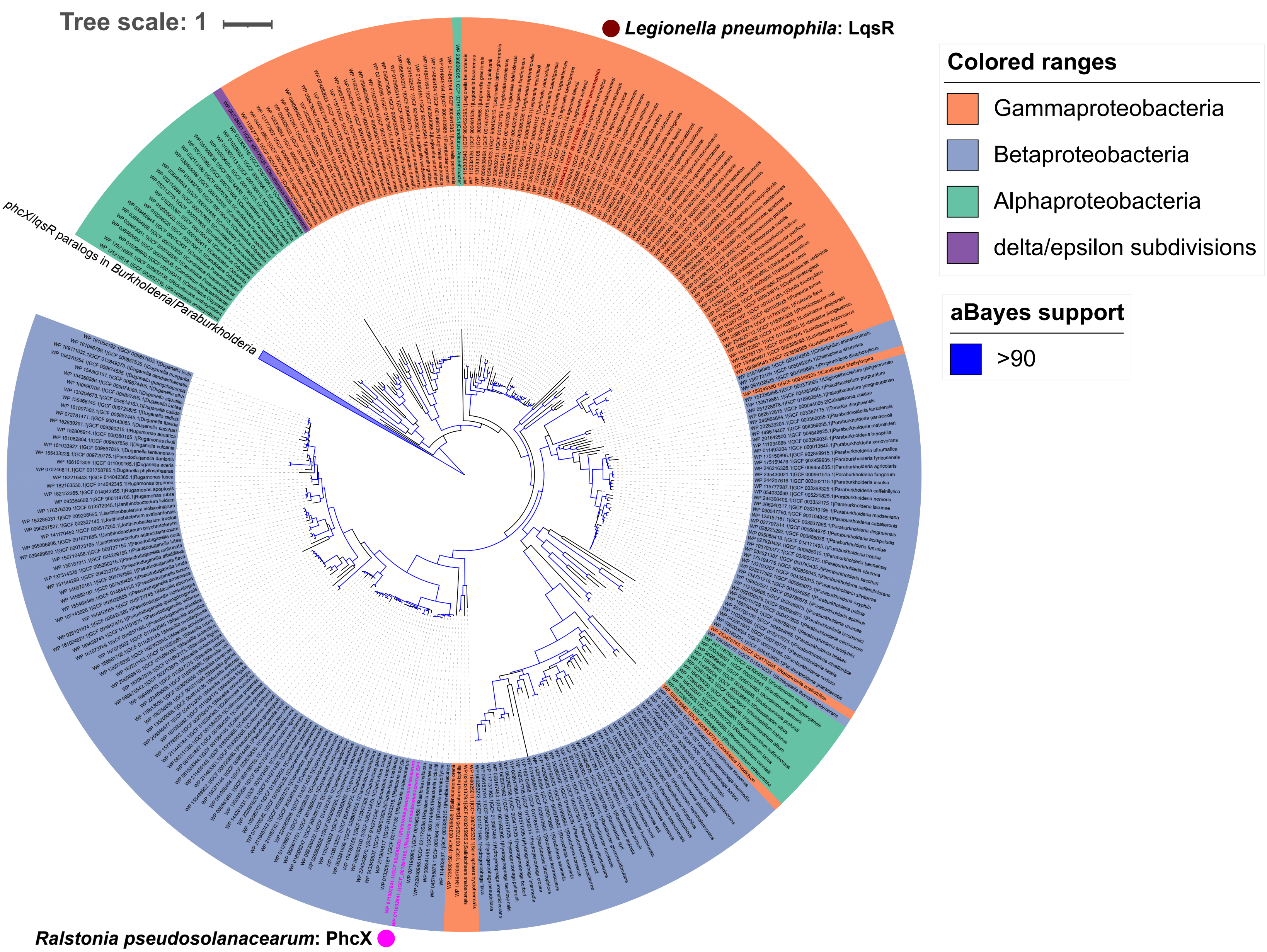

FIG S5. Phylogenetic analysis of PhcX, LqsR, and their homologs in representative Proteobacteria genomes. The maximum-likelihood phylogenetic tree was inferred by IQ-TREE v2.1.2 and the branch support was evaluated by the approximate Bayes (aBayes) test. Branches with aBayes support values greater than 90% were highlighted in blue. The tree was rooted between PhcX/LqsR orthologs in Proteobacteria and a clade of more distant paralogs in *Burkholderia*/*Paraburkholderia* (collapsed in the tree). The tree showed that the evolutionary relationships between PhcX, LqsR, and their homologs are largely consistent with the species phylogeny of Proteobacteria, further supporting the orthology between *R. solanacearum* PhcX and *L. pneumophila* LqsR. Specifically, *R. solanacearum* PhcX is most closely related to its orthologs in other Beta-proteobacteria, while the orthologs in Gamma-proteobacteria (including LqsR in *L. pneumophila*), Delta-proteobacteria, and Alpha-proteobacteria are more distantly related, with the exception of a few minor, putative horizontal gene transfer events.
