# Supplementary material for: PhcX Is a LqsR-family response regulator that contributes to Ralstonia solanacearum virulence and regulates multiple virulence factors
Source: mBio. 2023 Oct 3;14(5):e02028-23. doi: 10.1128/mbio.02028-23 (PMC10653808; doi:10.1128/mbio.02028-23)
Supplement: Table S1 — Mutations in EPS-deficient mutants; strains, plasmids, and PCR primers. [file mbio.02028-23-s0006.docx]

**Table S1A Mutations in EPS-deficient mutants**

| Mutants | gene | Product | Mutation |
| --- | --- | --- | --- |
| EP1_1_11 | *AC251_RS04265*,  *pilT* | type IV pilus twitching motility protein PilT | G403T |
| EP1_1_23_1 | *AC251_RS04265*,  *pilT* | type IV pilus twitching motility protein PilT | A587G |
|  | *AC251_RS03885* | response regulator | G83A |
| EP1_1_23_2 | Mutations are the same as EP1_1_23_1 | | |

**Table S1B** Strains and plasmids used in this study

| Name | Relevant characteristics | Source or reference |
| --- | --- | --- |
| ***R. solanacearum* strains** | | |
| EP1 | Wild-type strain, phylotype I, Rif^r^ | (1) |
| GMI1000 | Wild-type strain, phylotype I, Rif^r^ | (2) |
| Δ*EphcX* | *phcX*-deleted mutant of EP1, Rif^r^, Gm^r^ | This study |
| Δ*EphcA* | *phcA*-deleted mutant of EP1, Rif^r^ ,Gm^r^ | This study |
| *EphcX*-comp | Δ*EphcX* harboring pBBR1-*phcX*_EP1_, Rif^r^, Gm^r^, Km^r^ | This study |
| Δ*GphcX* | *phcX*-deleted mutant of GMI1000, Rif^r^, Gm^r^ | This study |
| *GphcX*-comp | Δ*GphcX* harboring pBBR1-*phcX*_GMI1000_, Rif^r^, Gm^r^ , Km^r^ | This study |
| ***Escherichia coli* strain** | | |
| DH5α | F-φ80d lacZΔM15 Δ(lacZYA-argF) U169 end A1 recA1 hsdR17 (rk-, mk+) supE44λ- thi-1 gyrA96relA1 phoA | TransGen Biotech |
| **Plasmids** |  |  |
| pK18*mobsac*B | Gm^r^, *sacB*-based gene replacement vector (3) |  |
| pBBR1MCS2 | Km^r^, broad-host-range cloning vector (4) |  |
| pBBR1-*phcX* | pBBR1MCS2 containing native *phcX* and the likely native promoter for *phcX* | This study |

**Table S1C** PCR primers used in this study

| **Primer name** | **Primer sequence (5’-3’)** |
| --- | --- |
| **For deletion** |  |
| EphcX-1 | CGGGAAGCTCACTCCACTAC |
| EphcX-2 | tgtgcgtccatgGCATTGTTCGACATGCCGTC |
| EphcX-3 | aacaatgcCATGGACGCACACCGTGG |
| EphcX-4 | agtcctttgtcgaaagcTGGCGGCGTTGTGACAAT |
| EphcX-5 | ccaGCTTTCGACAAAGGACTGGCT |
| EphcX-6 | GACATCAACAGCGAGTTCGG |
| GphcX-1 | CGGGAAGCTCACTCCACTAC |
| GphcX-2 | tgtgcgtccatgGCATTGTTCGACATGCCGTC |
| GphcX-3 | aacaatgcCATGGACGCACACCGT |
| GphcX-4 | agtcctttgtcgaaagcTGGCGGCGTTGTGACAAT |
| GphcX-5 | ccaGCTTTCGACAAAGGACTGG |
| GphcX-6 | GACATCAACAGCGAGTTCGG |
| EphcA-1 | TGAAGGGCGTGCTGGGTTA |
| EphcA-2 | tccatgCGTCATGTGCATCTTTTCCGC |
| EphcA-3 | agatgcacatgacgCATGGACGCACACCGTGG |
| EphcA-4 | TGGCGGCGTTGTGACAAT |
| EphcA-5 | aaattgtcacaacgccgccaCAGGTGGTGGCCGAATACAC |
| EphcA-6 | CCAGCGAAGAGCGTCACAT |
| Gen-F | TCCAGAACCTTGACCGAACG |
| Gen-R | TAGGTGGCTACGTCTCCGAA |
| **For complementation** |  |
| CphcX-F | gtcgacggtatcgataagcttAAGAACGACCCGAAATCCCC |
| CphcX-R | tcccccgggctgcaggaattcCCATCATTCGTCCCATGCTC |
| MCS-F | CAGGGTTTTCCCAGTCACGA |
| MCS-R | ATGCTTCCGGCTCGTATGTT |
| **For RT-qPCR** |  |
| Q-recA-F | CACCGAAGCGTAGAACTTGA |
| Q-recA-R | CCAACTGCCTGGTGATCTT |
| Q-lecM-F | ATGGCTCAGCAAGGTGTATTC |
| Q-lecM-R | TGGGTGTTCGCTGCATTT |
| Q-fliC-F | ACCAACCTGGACAAGGAATA |
| Q-fliC-R | GCTTGTTGCCGTTGTAGTTG |
| Q-pehB-F | TCACACCAGAATGACCTTTCA |
| Q-pehB-R | GACCGGATGGTGAAGGTATAA |
| Q-cheR-F | CGACTTCCGCCAGATCAA |
| Q-cheR-R | GGGTCGGCTTGTCGAAATA |
| Q-cheW-F | GCAGAGCCAGCAGATCAA |
| Q-cheW-R | GATGTCCACCAGGATCAACA |
| Q-cheY-F | CGATCCTGATGCTGACCAC |
| Q-cheY-R | AGACCGTCCGGATCGAAA |
| Q-cheA-F | CTCGATTCCGAAGGCAAGAA |
| Q-cheA-R | CACCTTGCGGTAATTGGTTTC |
| Q-Rsp1363-F | CAGACCATGAGCACGATCAA |
| Q-Rsp1363-R | CGTTGAGCGCCAGGATATT |
| Q-motA-F | TTTCCAACTATCCCGCCATC |
| Q-motA-R | CTTCGATCTGGAACGGATTCA |
| Q-flgG-F | TACACGCTGCTCAAGAAGAC |
| Q-flgG-R | CGACAGGTTGCCGAACA |
| Q-flgK-F | GACGGCCAGTACAACATCTAC |
| Q-flgK-R | GCTGCGTCGGATCGTATT |
| Q-flgL-F | GACTACACCAGCGCCATTT |
| Q-flgL-R | TGATGTACTGGAACAACGACAG |
| Q-flgN-F | TCGAACTGGAACGCATCAC |
| Q-flgN-R | CATCCGAGCGCAGGTATTT |
| Q-hrcC-F | CATCATCGACATCAGCGAGAA |
| Q-hrcC-R | GGTTGTTGAGCATGGTGTTG |
| Q-hrpB-F | GATTTCGACCGTGCGTATCA |
| Q-hrpB-R | AAGCCTTCTTCCGCTTCTTC |
| Q-hrpG-F | GCATCGATCATCAGCAGACT |
| Q-hrpG-R | AATCCATCCAGCTTCGCTTAT |
| Q-hrpY-F | GAAACCCACCACGACGAA |
| Q-hrpY-R | TTGGTATTGCGCCTGGAA |
| Q-popA-F | CAACGACCCGAGCAAGAA |
| Q-popA-R | GCATCGGATCCTGGTTGTT |
| Q-epsR-F | CGAGGATTTCATATTGCCGTTG |
| Q-epsR-R | GCGGCGCTGGAATTAGTA |
| Q-rasI-F | GTCGCTATCGCTACAAGGTATT |
| Q-rasI-R | GCGATCAAATGCTTCGTGTC |
| Q-solI-F | TTCGAGCGCGACCAATAC |
| Q-solI-R | GAACACGTCCTTGAGCAGATAG |
| Q-narG-F | TCCGAAGAGGTCAGCTACAA |
| Q-narG-R | AGCGGTGGTCCTGATAGAA |
| Q-narH-F | TACTACGAGCCCTTCACCTAC |
| Q-narH-R | CATTCGATCTTCTCCATGGTCTT |
| Q-ccoN-F | ACGCCCAGACGTTCAATTAC |
| Q-ccoN-R | CAGTTGCGGCCAGATCAA |
| Q-CcoO-F | GACTGGCATCGTATCCATCTG |
| Q-CcoO-R | TCGCTTCGATGTCATGGTTATC |
| Q-CcoP-F | TTCTCAACGCCGCATCAA |
| Q-CcoP-R | ACAGCAGGAACATCCACATC |
| **For RT-PCR** |  |
| phcQ-phcR1_F | CTCCACCGTGTCCTCGATTT |
| phcQ-phcR1_R | GGATCATGCAGTCGTTCGGA |
| phcQ-phcR2_F | CCAATGTCTCGTCGTCCACA |
| phcQ-phcR2_R | GCTCGACACCTATCCGTTCA |
| phcR-phcS_F | CTGCTCGTCGTCCACATACA |
| phcR-phcS_R | GAAGTAAACCGCCAGTTGCC |
| phcS-phcB_F | GGTCAGCATCATCAGCGAGA |
| phcS-phcB_R | CAGCGAGGAAACCCTCGAAT |
| phcB-phcX1_F | CCGCGAACCTGCTGACCCTG |
| phcB-phcX1_R | GAGGCCTACGATGCCCCCGA |
| phcB-phcX2_F | CTTCCCAGTCCTGGACGAAC |
| phcB-phcX2_R | CCTGATGGTGATCGAGACCG |

1. **Li P, Wang D, Yan J, Zhou J, Deng Y, Jiang Z, Cao B, He Z, Zhang L.** 2016. Genomic analysis of phylotype I strain EP1 reveals substantial divergence from other strains in the *Ralstonia solanacearum* species complex. Front Microbiol 7:1719.

(2) **Salanoubat M, Genin S, Artiguenave F, Gouzy J, Mangenot S, Arlat M, Billault A, Brottier P, Camus JC, Cattolico L, Chandler M, Choisne N, Claudel-Renard C, Cunnac S, Demange N, Gaspin C, Lavie M, Moisan A, Robert C, Saurin W, Schiex T, Siguier P, Thebault P, Whalen M, Wincker P, Levy M, Weissenbach J, Boucher CA.** 2002. Genome sequence of the plant pathogen *Ralstonia solanacearum*. Nature 415:497-502.

1. **Andreas Schäfer, Andreas Tauch, Wolfgang Jäger, Jörn Kalinowski, Georg Thierbach, Alfred Pühler.** 1994. Small mobilizable multi-purpose cloning vectors derived from the *Escherichia coli* plasmids pK18 and pK19: selection of defined deletions in the chromosome of *Corynebacterium glutamicum*, Gene 145:69-73.

(4) **Kovach ME, Elzer PH, Hill DS, Robertson GT, Farris MA, Roop RM 2nd, Peterson KM.** 1995. Four new derivatives of the broad-host-range cloning vector pBBR1MCS, carrying different antibiotic-resistance cassettes. Gene 166:175-6.
